# Genetic diversity of a short‐ranged endemic terrestrial snail

**DOI:** 10.1002/ece3.10785

**Published:** 2023-11-28

**Authors:** Lachlan J. Gretgrix, Orsi Decker, Peter T. Green, Frank Köhler, Adnan Moussalli, Nicholas P. Murphy

**Affiliations:** ^1^ Department of Environment and Genetics, School of Agriculture, Biomedicine and Environment La Trobe University Melbourne Victoria Australia; ^2^ Bavarian National Park Nationalparkverwaltung Bayerischer Wald Grafenau Germany; ^3^ Australian Museum Sydney New South Wales Australia; ^4^ Museums Victoria Carlton Victoria Australia

**Keywords:** *Austrochloritis kosciuszkoensis*, cryptic, population structure, SNPs, terrestrial snails

## Abstract

The factors that influence population structure and connectivity are unknown for most terrestrial invertebrates but are of particular interest both for understanding the impacts of disturbance and for determining accurate levels of biodiversity and local endemism. The main objective of this study was to determine the historical patterns of genetic differentiation and contemporary gene flow in the terrestrial snail, *Austrochloritis kosciuszkoensis* (Shea & O. L. Griffiths, 2010). Snails were collected in the Mt Buffalo and Alpine National Parks in Victoria, in a bid to understand how populations of this species are connected both within continuous habitat and between adjacent, yet separate environments. Utilising both mitochondrial DNA (mtDNA) and single nucleotide polymorphism (SNP) data, the degree of population structure was determined within and between sites. Very high levels of genetic divergence were found between the Mt Buffalo and Alpine snails, with no evidence for genetic exchange detected between the two regions, indicating speciation has possibly occurred between the two regions. Our analyses of the combined mtDNA and nDNA (generated from SNPs) data have revealed patterns of genetic diversity that are consistent with a history of long‐term isolation and limited connectivity. This history may be related to past cycles of changes to the climate over hundreds of thousands of years, which have, in part, caused the fragmentation of Australian forests. Within both regions, extremely limited gene flow between separate populations suggests that these land snails have very limited dispersal capabilities across existing landscape barriers, especially at Mt Buffalo: here, populations only 5 km apart from each other are genetically differentiated. The distinct genetic divergences and clearly reduced dispersal ability detected in this data explain the likely existence of at least two previously unnamed cryptic *Austrochloritis* species within a 30–50 km radius, and highlight the need for more concentrated efforts to understand population structure and gene flow in terrestrial invertebrates.

## Graphical abstract

We evaluated the potential response of a highly immobile native land snail species, *Austrochloritis kozsciouskoensis*, to future disturbance events. Our study aimed to determine the historic patterns of genetic differentiation and gene flow of *A. kozsciouskoensis* populations in the Mt Buffalo and Alpine (along Kiewa River) National Parks in Victoria, and we found that there were signs of limited gene flow between populations within each region, with no evidence of genetic exchange present between the two regions. We have concluded that the Mt Buffalo and Kiewa populations likely represent individual cryptic species.

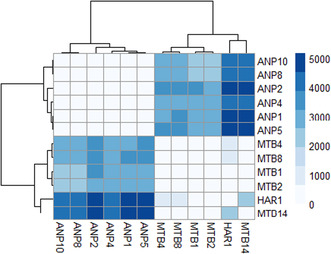

## 1 INTRODUCTION

Short‐ranged endemic (SRE) species are described as occupying ranges of less than 10,000 km^2^ often across heavily fragmented environments, and are generally habitat specialists with low reproductive rates and poor dispersal ability (Harvey, 2002; Mason et al., 2018). These attributes make SREs extremely susceptible to disturbances and climate change (Harvey et al., 2011), which is why they are typically included on the IUCN list as one of the most vulnerable trait groups due to climate change (Foden et al., 2009). In Australian ecosystems, SREs are predominantly represented by flightless terrestrial species such as millipedes, isopods, amphipods, spiders, velvet worms and snails (Harvey, 2002). In nature SRE species have a proclivity to specialise in distinct habitats, without the need of rapidly recolonising their distribution. This process leads to naturally isolated populations, resulting in the frequent occurrence of allopatric speciation (Harvey, 2002; Mason et al., 2018), often without morphological change, resulting in cryptic species (Bickford et al., 2007; Sáez & Lozano, 2005). The commonly found relationship between SREs and cryptic species stems from the fragmentation of niche habitats that leads to isolated populations that genetically diverge over hundreds of thousands of years, while often still retaining similar phenotypic traits (Emata & Hedin, 2016). Short‐range, cryptic species also provide a particular challenge regarding accurately measuring biodiversity, and for understanding the true impact of habitat fragmentation and large‐scale disturbances (Bickford et al., 2007). Genetic studies of population structure and gene flow can help bridge this knowledge gap by improving our knowledge of the diversity within species, as well as identifying species diversity that has gone previously unnoticed.

Terrestrial gastropods are a clear example of SRE taxa for which knowledge is limited (Harvey, 2002). Land snails generally have limited dispersal ability (Parkyn & Newell, 2013), with some known to move no more than 350 m from birth to breeding age (Clark & Richardson, 2002). Many land snail species are also restricted to a single habitat type, particularly those dependent on moist environments such as mesic, or rainforest habitats (Smith, 1979), as well as limestone specialists (Haskell & Pan, 2013). Conversely, a number of snail species have become global invaders (Blacket et al., 2016), and it is suggested that some larger snail species like *Hedleyella falconeri* have increased dispersal ability that may enable gene flow between populations in spite of habitat fragmentation (Murphy, 2002).

The purpose of this study was to increase our understanding of SRE organisms, by examining gene flow and population structure in the terrestrial land snail, the Kosciuszko Bristle Snail (*Austrochloritis kosciuszkoensis* Shea and O. L. Griffiths, 2010). The genus *Austrochloritis* encompasses 34 described species, distributed throughout rainforests, wet sclerophyllous forests and woodlands in south‐eastern Australia (Köhler et al., 2020; Shea & Köhler, 2019; Stanisic et al., 2010). These species are narrowly distributed throughout fragmented forest environments with most described as SREs (Shea & Köhler, 2019), and as such, a number of these species, including *A. kosciuszkoensis*, were identified for priority research following the recent 2019/2020 Australian megafires (Legge et al., 2021), in which over 10 million hectares were burnt country‐wide, 81% identified as native forests (Davey & Sarre, 2020; Saunders et al., 2021), and it was estimated over 240 trillion invertebrates were killed (Dickman, 2021) as a result of this unprecedented event (Boer et al., 2020).

Both mitochondrial DNA (mtDNA) and single nucleotide polymorphism (SNP) approaches were utilised in this study. Studies using mtDNA have long been the mainstay for terrestrial SREs and have proven to be critical for assessing long‐term evolutionary patterns, such as allopatric differentiation driven by low dispersal ability in groups such as amphipods (Menz et al., 2016) and beetles (Papadopoulou et al., 2008), and for examining the relationships between landscape and population structure (Garrick et al., 2012). In addition, mtDNA studies have proven critical for the detection of cryptic species in SREs (Raphalo et al., 2021). On the other hand, SNP analyses have proven effective in detecting fine‐scale population genetic differentiation and can provide a more detailed analysis of gene flow between populations (McGaughran et al., 2019). However, the species‐specific nature of obtaining and utilising SNPs limits the application of SNP‐based studies on non‐model species (Ogden et al., 2012) like SREs. To fill this gap, our study investigates the potential levels of genetic diversity present in short‐range endemic species by an evaluation of our model species, *A. kosciuszkoensis*, within a small part of its range. Given the likely poor dispersal of this species and the altitude and habitat changes within its distribution, we hypothesised that snails form genetically distinct allopatric populations between catchments. In contrast, it was expected that the continuous habitat within regions would lead to less perceptible population structure within regions in comparison to between regions.

## 2 MATERIALS AND METHODS

### 2.1 Site and sampling information

Our study was undertaken on local populations of *A. kosciuszkoensis* inhabiting a small portion of the species' range, including the Alpine National Park (hereafter Alpine NP) and the neighbouring, albeit isolated, Mt Buffalo National Park (hereafter Mt Buffalo NP). Mt Buffalo NP encompasses a mountain plateau with altitudes ranging from ~300 to ~1700 m with a variety of alpine, subalpine and mesic forest habitats distributed throughout the region. The area of the Kiewa and Ovens Rivers within the Alpine NP covers a similar range of altitudes, ~300 to ~1200 m, but offers a more continuous stretch of mesic forest habitats.

**FIGURE 1:**
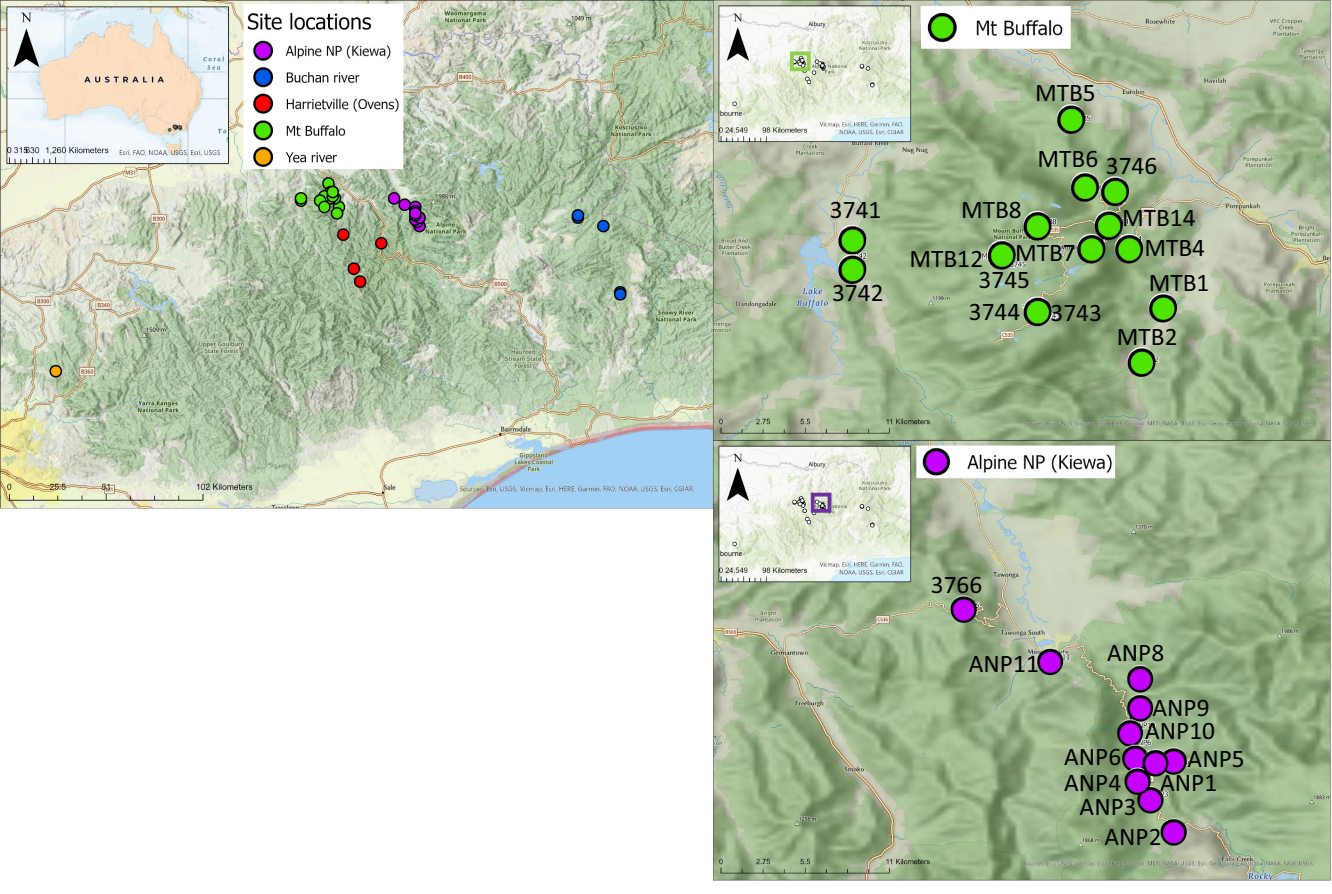
Map of *Austrochloritis kosciuszkoensis* collection sites in regional Victoria, Australia. Green = Mt Buffalo NP, Purple = Alpine NP/Kiewa River, Red = Harrietville/Ovens River, Blue = Buchan River, and Orange = Yea River.

Specimens of *A. kosciuszkoensis* were collected from moist forest habitats in Mt Buffalo NP and the adjacent regions of the Alpine NP (Taungurung and Gunaikurnai Country) along the Kiewa and Ovens Rivers (Figure 1) in Victoria, Australia. Sampling was undertaken at 25 sites (13 sites at Mt Buffalo and 12 sites at Kiewa); however, due to the rarity of *A. kosciuszkoensis*, individuals were only found at 19 of these sites (Figure 1). Within the Alpine NP, two distinct groups of sites from different catchments were sampled, 11 from the Kiewa catchment, and one from the neighbouring Ovens catchment. The Mt Buffalo sites also fall along the Ovens catchment, although from separate sub‐catchments along the Buckland River. In addition to the samples collected, 18 *A. kosciuszkoensis* mtDNA sequences generated from previously unpublished studies from the surrounding region were also included (Table 1), in addition to MN510625 (*A. kosciuszkoensis*), MN512673 (*A. stanisici*), MN512684 (*A. abrotonus*), and MN510584 (*A. kanangra*) from Genbank (https://www.ncbi.nlm.nih.gov/genbank/).

**TABLE 1:** Site information and individual snail counts for mtDNA and SNP analyses.

Site	Region	Catchment	Elevation (m)	Longitude	Latitude	mtDNA	SNP
MTB1	Mt Buffalo NP	Buckland River	319	146.86	−36.76	9	9
MTB2	Mt Buffalo NP	Buckland River	345	146.85	−36.79	13	12
MTB4	Mt Buffalo NP	Eurobin Creek	473	146.84	−36.72	13	12 (13)
MTB5	Mt Buffalo NP	Buffalo Creek	665	146.81	−36.65	5	‐
MTB6	Mt Buffalo NP	Buffalo Creek	667	146.82	−36.69	3	‐
MTB7	Mt Buffalo NP	Crystal Brook	1314	146.82	−36.72	3	‐
MTB8	Mt Buffalo NP	Buffalo Creek	1198	146.79	−36.71	13	12 (13)
MTB12	Mt Buffalo NP	Crystal Brook	1372	146.77	−36.73	3	‐
MTB14	Mt Buffalo NP	Eurobin Creek/Buffalo Creek	911	146.83	−36.71	3	2
HAR1	Harrietville/Ovens	Dickinson Creek/Miners Creek	1114	147.06	−36.93	9	8 (9)
ANP1	Alpine NP/Kiewa	Rocky Valley Creek/Pretty Valley Creek	696	147.23	−36.81	5	5
ANP2	Alpine NP/Kiewa	Turntable Creek	1197	147.24	−36.85	1	1
ANP3	Alpine NP/Kiewa	Pretty Valley Creek	1010	147.23	−36.83	3	‐
ANP4	Alpine NP/Kiewa	Rocky Valley Creek	762	147.22	−36.82	11	11
ANP5	Alpine NP/Kiewa	Pretty Valley Creek	759	147.24	−36.81	6	6
ANP6	Alpine NP/Kiewa	Kiewa River East Branch	721	147.22	−36.80	1	‐
ANP8	Alpine NP/Kiewa	Kiewa River East Branch	464	147.22	−36.76	6	5
ANP9	Alpine NP/Kiewa	Kiewa River East Branch	565	147.22	−36.78	1	‐
ANP10	Alpine NP/Kiewa	Kiewa River East Branch	627	147.22	−36.79	6	5
ANP11	Alpine NP/Kiewa	Mount Beauty Pondage	381	147.17	−36.75	1	‐
*A. sp* NMV	Yea River	Yea River	658	145.52	−37.53	1	‐
3746	Mt Buffalo NP	Buffalo Creek	810	146.83	−36.69	1	‐
3742	Mt Buffalo NP	Lake Buffalo	286	146.68	−36.73	1	‐
3744	Mt Buffalo NP	Skeleton Gully	1478	146.79	−36.76	1	‐
3741	Mt Buffalo NP	Buffalo River	329	146.68	−36.72	1	‐
3766	Alpine NP/Kiewa	German Creek	851	147.12	−36.72	1	‐
3748	Harrietville/Ovens	Mount Selwyn Creek	909	146.93	−37.05	1	‐
3747	Harrietville/Ovens	Mount Selwyn Creek	909	146.93	−37.05	1	‐
3749	Harrietville/Ovens	Mount Selwyn Creek	1093	146.96	−37.11	1	‐
3750	Harrietville/Ovens	Clear Creek	440	146.88	−36.89	1	‐
3756	Buchan River	Old Place Creek	1214	148.19	−37.16	1	‐
3757	Buchan River	Old Place Creek	1167	148.19	−37.17	1	‐
3753	Buchan River	Bulley Creek	1287	148.11	−36.85	1	‐
3754	Buchan River	Bulley Creek	1287	148.11	−36.85	1	‐
3767	Buchan River	Buckwong Creek	1536	147.99	−36.81	1	‐
3752	Buchan River	Buckwong Creek	1526	147.99	−36.80	1	‐
3745	Mt Buffalo NP	Crystal Brook	1382	146.77	−36.73	1	‐
3743	Mt Buffalo NP	Skeleton Gully	1478	146.79	−36.76	1	‐

*Note*: Numbers in brackets represent counts prior to samples being removed.

### 2.2 DNA extraction

Whole snail samples were held in ethanol at room temperature prior to DNA extractions, which were undertaken from the foot muscle of each specimen via dissection using a DNeasy Blood and Tissue kit (QIAGEN), following the manufacturers' instructions. A 658 bp fragment of the Mitochondrial gene Cytochrome *c* oxidase (CO1) was selected and amplified using the primers LCO1490 and HCO2198 (Folmer et al., 1994). PCRs were undertaken in 25 μL reactions; 12.5 μL of GoTaq green mix, 9.5 μL of Milli‐Q water, 0.50 μL of the LCO1490 and HCO2198 primers respectively, and 2 μL of sample DNA. PCR protocol consisted of an initial denaturation period at 95°C for 5 min, followed by 35 cycles of 95°C for 30 s, 47°C for 30 s and 72°C for 45 s, followed by one final extension of 72°C for 5 min. Samples were sent to the Macrogen Bioinformatics centre (https://dna.macrogen.com/) for Sanger sequencing. Samples were also sent to Diversity Arrays Technology (DArT) (https://www.diversityarrays.com/) in Canberra for DArT sequencing (DArTseq) (Kilian et al., 2012). The DArTseq methodology incorporates a reduced representation sequencing method, similar to double‐digest restriction‐site associated DNA sequencing, that involves using restriction enzymes (PstI and SphI‐HF) to select tens of thousands of loci across a nuclear genome for SNP markers in order to test for the level of variation existing between genetic sequences (Anderson et al., 2017; Bonin et al., 2008; Kilian et al., 2012).

### 2.3 Statistical analysis

#### 2.3.1 mtDNA: establishment of lineages

Mitochondrial DNA sequences were aligned and edited using Geneious Prime Vers. 2021.2.2. (Kearse et al., 2012). Geneious Prime Vers. 2021.2.2. was also used to produce a haplotype identity matrix. The Geneious addon PAUP* (Wilgenbusch & Swofford, 2003) was used to determine the ideal best‐fit model of sequence evolution (TN93+I+G submodel of GTR+I+G) via the Akaike Information Criterion (AIC) (Akaike, 1974). The ML (Maximum‐Likelihood) topology was constructed with 1000 bootstrap replicates using the Geneious addon Phyml (Guindon et al., 2010) and edited in FigTree v1.4.4 (http://tree.bio.ed.ac.uk/software/figtree/). The Geneious addon RAxML (Stamatakis, 2014) was used to construct a tree using the GTR+I+G model and data partitioned by codon position with 1000 bootstrap replicates. The Geneious addon MrBayes (Huelsenbeck & Ronquist, 2001) was used to construct a tree supported by Bayesian inference, the GTR substitution model was used, with the data partitioned by codon position, and a chain length of 1,100,000, subsampling frequency of 200, and burn‐in length of 100,000.

To examine the potential for speciation among lineages, a maximum likelihood species delineation tree was also produced using the Exelixis Lab bPTP web server (https://species.h‐its.org/ptp/), by running the dataset for 500,000 MCMC generations, a burn‐in of 0.10, thinning of 100 and a seed of 123 (Zhang et al., 2013). Convergence was checked via the produced trace file, using tracer v1.7.2 (Rambaut et al., 2018). In addition, the program ASAP (Puillandre et al., 2021) was used to partition samples into “species” groups, while an uncorrected *p*‐distance matrix was produced using the program ABGD (Puillandre et al., 2012).

#### 2.3.2 mtDNA: population structure within lineages

Within lineage nucleotide divergence and diversity and haplotype diversity were calculated in DnaSP v61203 (Rozas et al., 2017) and population pairwise *F*_ST_ values were calculated in Arlequin ver 3.5.2.2 (Excoffier & Lischer, 2010). PopART v1.7 was used to produce a Median‐Joining Network (Clement et al., 2002; Leigh & Bryant, 2015). To identify any fine‐scale deviations that could be masked by whole data analyses, separate Mt Buffalo and Kiewa haplotype networks were also created.

#### 2.3.3 SNP: establishment of lineages

Ninety‐one samples were sent to DArT for SNP sequencing (Table 1). Of these, 2 samples did not meet the necessary quantity and quality requirements (20 μL of DNA at 50–100 ng/μL and >1.6 ratio of absorbance at 260 and 280 nm). An additional sample was removed during filtering due to missing data. 76,620 SNP markers were produced by the remaining 88 samples.

Single nucleotide polymorphism data was further filtered using the “dartR” package (Gruber et al., 2018) in “R” version 4.1.1 (R Core Team, 2013) by: removing monomorphic SNPs using the function gl.filter.monomorphs, filtering with a threshold for equivalent accuracy of 0.999 using the function gl.filter.reproducibility (threshold = 0.999), removing loci missing >20% individuals using the function gl.filter.callrate (method = “loc”, threshold = 0.85), removing individuals missing >15% loci using the function gl.filter.callrate (method = “ind”, threshold = 0.8), filtering based on minor allele frequency using the function gl.filter.maf (threshold = 0.03) (at least three counts with a minor allele) and filtering markers found on the same sequencing tag using the function gl.filter.secondaries. After filtering, the full 88 sample dataset contained 15,654 SNPs. Due to the high quantity of fixed differences produced between the Mt Buffalo sites and Kiewa sites, two separate datasets were made to determine fine‐scale patterns within regions. The 47 Mt Buffalo sample dataset contained 12,175 SNPs, and the 33 Kiewa sample dataset contained 2309 SNPs.

Population structure was investigated using multiple approaches. First, the “dartR” package was used to produce a full dataset PCA (Principal Component Analysis), a visual representation of samples in genomic space, using the function ggplot. The package adegenet (Jombart, 2008) was then used to perform a “DAPC” (Discriminant Analysis of Principle Components), a multivariate method of grouping sites into clusters based on genetic similarities, using BIC (Bayesian Information Criterion) in order to determine the number of populations present (Schwarz, 1978). The number of clusters was determined by first selecting the number of Principal Components (PCs) to retain that allows for 100% of cumulative variance to be explained, and then select the number of clusters that produced the lowest BIC value, as described by Jombart and Collins (2015), using the functions scaleGen, find.clusters, table.value, dapc and scatter. Additional analysis of clusters was performed using the program Structure (Pritchard et al., 2000), with 10 independent runs for *k* values 1–10, a burn‐in of 100,000 and 500,000 MCMC replicates. Structure Harvester (Earl & VonHoldt, 2012) was used to calculate the optimal number of genetic clusters (*k*‐value), and CLUMPAK (Kopelman et al., 2015) was used to visualise the data as a Structure plot with the optimal *k*‐value.

A fixed difference analysis was performed with the use of the “dartR” and “pheatmap” packages (Kolde & Kolde, 2015) to produce heat maps and matrices, representing the absolute allele differences between sites, using the functions gl.fixed.diff and gl.plot.heatmap. A single “fixed difference” refers to when two sites share no alleles at a locus, with the accumulation of a multitude of fixed differences implying limited gene flow has occurred (Huber et al., 2016).

Given the large genetic differences apparent in the data, coalescent‐based species delimitation analysis was undertaken on the SNP dataset using Bayes Factor Delimitation (BFD*) (Leaché et al., 2014) implemented using SNAPP (Bryant et al., 2012) in BEAST2 v. 2.7.5 (Bouckaert et al., 2019). To reduce the computational resources required, 200 SNPs with at least 1 individual included from each site and at least 2 individuals from each cluster generated by Structure (total of 15 individuals included in dataset) were randomly selected from the fully filtered 15,654 SNP dataset. A path sampling analysis was undertaken to estimate and rank marginal likelihoods between 5 separate speciation models: 1: speciation is driven by catchments (Kiewa vs. Ovens (Mt Buffalo + Harrietville)), 2: speciation is driven by catchments and altitude (Kiewa vs. Harrietville vs. Mt Buffalo), 3: Structure clusters within Mt Buffalo are also separate species (Kiewa vs. Harrietville vs. Mt Buffalo1 vs. Mt Buffalo2), 4: Structure clusters within Kiewa are also separate species (Kiewa1 vs. Kiewa2 vs. Kiewa3 vs. Harrietville vs. Mt Buffalo), and 5: all Structure clusters are separate species (Kiewa1 vs. Kiewa2 vs. Kiewa3 vs. Harrietville vs. Mt Buffalo1 vs. Mt Buffalo2). For each speciation model, marginal likelihood estimates (MLE) were calculated using SNAPP, and path sampling analyses with 20 steps for 200,000 iterations after a 20% burnin and setting alpha to 0.3. Stationarity of all runs was checked and each step was run until ESS ≥200.

#### 2.3.4 SNP: population structure within lineages

Using the “hierfstat” package in R (Goudet, 2005), pairwise *F*_ST_ tests were run to determine the similarities among sites with >5 individuals using the functions genind2hierfstat, pairwise.WCfst and boot.ppfst. The “hierfstat” and “dartR” packages were also used to calculate population genetic diversity measures (Ho = observed heterozygosity, He = expected heterozygosity, Hs = observed genetic diversity, Fis = inbreeding coefficient, and AR = Allelic richness), using the functions genind2hierfstat, basic.stats, basicstats$n.ind.samp, basicstats$Ho, basicstats$Hs, basicstats$Fis, basicstats$overall and allelic.richness.

An “isolation‐by‐distance” test was performed with 1000 permutations via the “ade4” package in R (Chessel et al., 2004) using the function gl.ibd, plotting any existing correlations between genetic differentiation and geographic distance, measured as straight‐line distances between site locations within the Mt Buffalo and Kiewa datasets respectively.

Average migration rates and ±95% confidence intervals (1.96 × standard deviation) between MTB sites and Kiewa sites respectively were calculated using BAYESASS 3.0.4 (Wilson & Rannala, 2003). Both datasets were run for 10,000,000 iterations at a seed of 100, with a burn‐in of 1,000,000 and a sampling interval of 100. Values for the parameters: allele frequency (‐a), Inbreeding coefficients (‐f), and migration rate (‐m), were adjusted for both datasets to produce acceptance rates between 0.2 and 0.4 as suggested by the creators of the program (Wilson & Rannala, 2003). The Kiewa dataset values were adjusted to ‐a = 0.95, ‐f = 0.1 (default) and ‐m = 0.5, whereas the MTB dataset values were adjusted to ‐a = 0.90, ‐f = 0.05 and ‐m = 0.5. The program Tracer v1.7.2 (Rambaut et al., 2018) was used to confirm convergence has occurred. The MTB dataset was run again under the same conditions, the two outputs were trimmed then combined using LogCombiner (Bouckaert et al., 2014) and adjusted to a burn‐in of 3,200,000 to improve results.

## 3 RESULTS

### 3.1 mtDNA broadscale analyses

**FIGURE 2:**
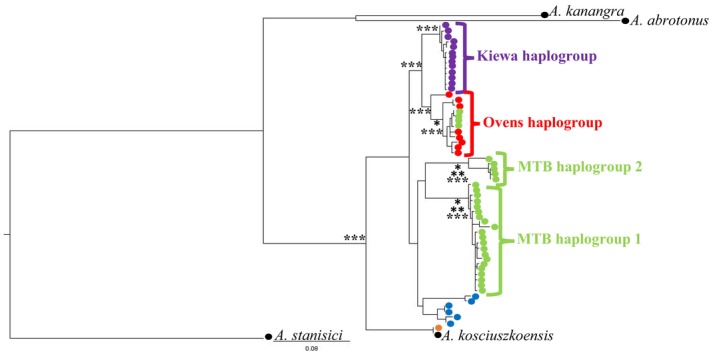
*Austrochloritis kosciuszkoensis* Maximum‐Likelihood haplotype tree depicting relationships based on 587 base pairs of the mitochondrial CO1 gene. “*” Represents ML bootstraps of 70% or over, “**” represents codon partition bootstraps of 70% or over and “***” represents Bayesian support of 90% or over. Purple node = Kiewa River, red node = Ovens River, green node = Mt Buffalo NP, orange node = Yea River, blue node = Buchan River and black node = outgroup *Austrochloritis* species.

A total of 115 mtDNA sequences were generated, among which 40 unique haplotypes were found. The Maximum Likelihood (ML) phylogram (Figure 2) was split into four distinct well‐supported haplogroups. The major haplogroups found were also present in both the Bayesian Inference (BI) and RAxML codon‐partitioned trees (not shown) and support from these analyses is presented alongside the ML tree. Of these four haplogroups, three include individuals from Mt Buffalo NP. There is strong nodal support for MTB haplogroup 1 (ML = 96.7, RAxML = 95, and BI = 100), which contained the majority of MTB samples. All Kiewa individuals formed a moderately supported haplogroup (ML = 69.4, RAxML = 54, and BI = 100). The Ovens individuals formed a moderately supported haplogroup (ML = 70.9, RAxML = 62, and BI = 98), but also included Mt Buffalo samples from sites MTB1 and MTB2, the geographically closest sites to the Ovens River, in addition to samples located along the headwaters of the Buckland River. An additional, small MTB haplogroup 2 (ML = 96.6, RAxML = 98, and BI = 100) was found that contains two samples from the altitudinally highest location sampled in Mt Buffalo, MTB12, and additional samples collected along the western (3741 and 3742), southern (3744) and eastern (3746) slopes of Mt Buffalo.

At the broader level, every haplotype was unique to its collection region. Forty mtDNA haplotypes were identified among 115 individuals; 24 haplotypes were unique to Mt Buffalo, 13 haplotypes were unique to Kiewa and 3 haplotypes were unique to the Ovens (Figure S1). Interestingly, the highest diversity was present on Mount Buffalo, with (Table S1) genetic distances between MTB haplogroup 1 and MTB haplogroup 2 ranged from 7.50% to 9.37%. The differences between the other haplogroups ranged from 7.16% to 9.03% between MTB haplogroup 1 and the Kiewa haplogroup, 6.30% to 8.52% between MTB haplogroup 1 and the Ovens haplogroup, 3.92% to 4.94% between the Ovens haplogroup and the Kiewa haplogroup and 6.64% to 7.16% between the Ovens haplogroup and MTB haplogroup 2.

When comparing uncorrected *p*‐distance values between haplotypes (Table S2), the highest diversity was present on Mount Buffalo, with values between haplotypes from MTB haplogroup 1 and MTB haplogroup 2 ranging from 0.079 to 0.10. The differences between the other haplogroups ranged from 0.075 to 0.096 between MTB haplogroup 1 and the Kiewa haplogroup, 0.068 to 0.090 between MTB haplogroup 1 and the Ovens haplogroup, 0.040 to 0.051 between the Ovens haplogroup and the Kiewa haplogroup and 0.070 to 0.075 between the Ovens haplogroup and MTB haplogroup 2. Unsurprisingly, very large population pairwise *F*_ST_ values (>0.9) were evident between haplogroups (Table S3) with the greatest difference recorded between haplogroups MTB haplogroup 2 and the Kiewa haplogroup (0.94).

The Maximum Likelihood Species Delineation Tree produced by bPTP (Figure S2) and the species partitions produced by ASAP suggest that the degree of between‐haplogroup differentiation is accordant to separate species (Table 2). Both methods agreed on the separation of Kiewa, Mt Buffalo Haplogroup 1 and Mt Buffalo Haplogroup 2 as separate species, while the bPTP method also separated the Ovens Haplogroup into separate species, splitting the Harrietville and Mt Buffalo haplotypes.

**TABLE 2:** Population differentiation and species delineation summary table.

Site	mtDNA analyses			SNP analyses		
	ASAP	bPTP	DAPC	Structure	Fixed difference	SNAPP
MTB8	A	A	B	A	A	B
MTB14	A	A	F	A	A	B
MTB4	A	A	F	A	A	B
MTB7^a^	A	A	‐	‐	‐	‐
MTB5^a^	A	A	‐	‐	‐	‐
MTB6^a^	A	A	‐	‐	‐	‐
MTB1	A	A	A	A	A	A
MTB2	A	A	D	A	A	A
MTB12^a^	A	A	‐	‐	‐	‐
MTB12^a^	B	B	‐	‐	‐	‐
ANP4	C	C	E	B	B	D
ANP10	C	C	E	B	B	E
ANP8	C	C	E	B	B	F
ANP6^a^	C	C	‐	‐	‐	‐
ANP2	C	C	E	B	B	D
ANP1	C	C	E	B	B	D
ANP9^a^	C	C	‐	‐	‐	‐
ANP3^a^	C	C	‐	‐	‐	‐
ANP5	C	C	E	B	B	D
ANP11^a^	C	C	‐	‐	‐	‐
HAR1	D	D	C	A	A^b^	C
MTB1	D	E	A	A	A	A
MTB2	D	E	D	A	A	A

*Note*: Letters represent the “species” or group that each method has assigned the samples by.

^a^ Represents sites that were present in the mtDNA data but was unable to be sequenced and included for the SNP data.

^b^ While the Harrietville site produced the least fixed differences between MTB sites, there were still relatively high numbers of fixed differences produced between HAR1 and some MTB sites.

### 3.2 mtDNA within‐regions analyses

**FIGURE 3:**
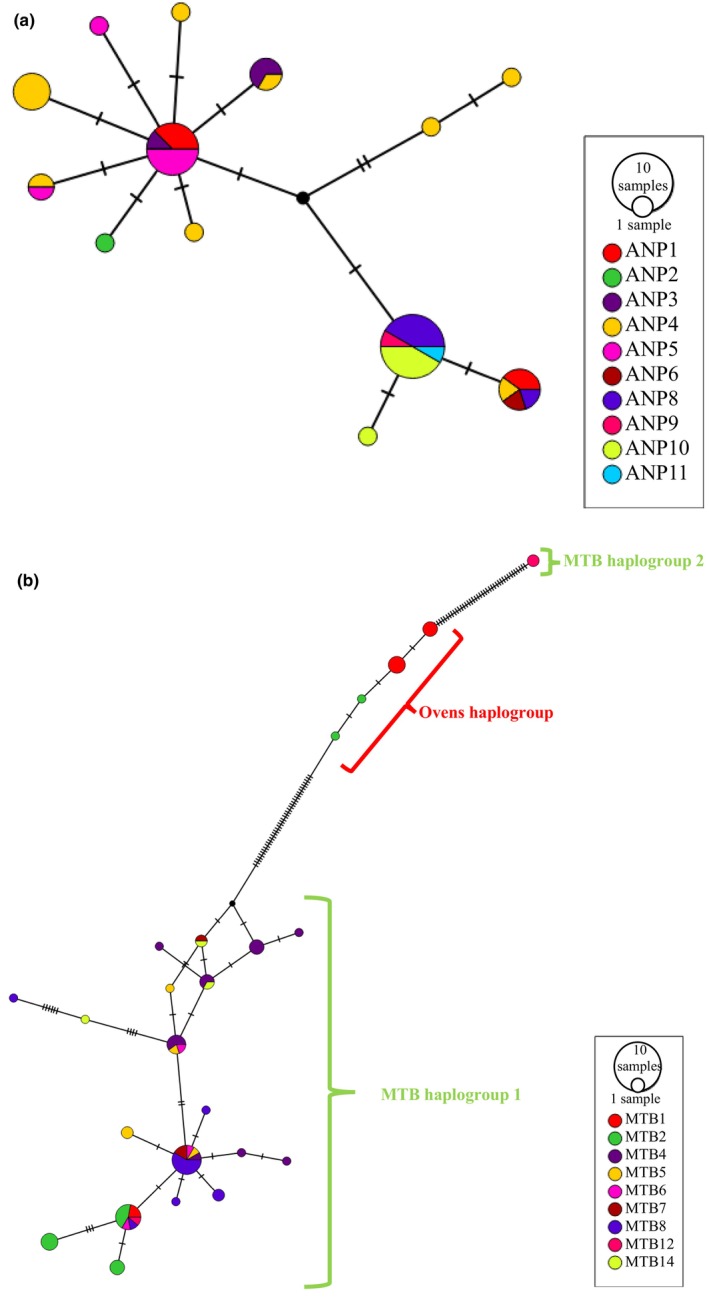
Median‐Joining Networks of *Austrochloritis* spp. mitochondrial haplotypes. Each circle represents a unique haplotype, circle size is proportionate to the number of individuals carrying the haplotype, black circles represent unsampled haplotypes, lines denote the number of base pair differences between haplotypes and colours represent the NP/region the samples belong to. (a) Only Kiewa haplotypes. (b) Only MTB haplotypes.

Within both the Kiewa and MTB regions, haplotypes were shared between sites that were geographically close (Figure 3a,b). Within the Kiewa network (Figure 3a), one of the most common haplotypes was found at 4 sites, with the greatest distance between sites sharing haplotypes being only 4 km between ANP11 and ANP8. Site ANP4 produced the greatest haplotypic diversity; five of these eight haplotypes were unique to that site (Figure 3a); however, this site was also the most successful Kiewa collection site with the most specimens collected from any individual Kiewa site (11).

Among the MTB sites, three sites have haplotypes from more than one haplogroup (Figure 3b), as shown by the ML topology. MTB haplogroups 1 and 2 both had individuals collected from site MTB12, while haplotypes belonging to MTB haplogroup 1 and the Ovens haplogroup are found at sites MTB1 and MTB2. Within the main MTB haplogroup, the maximum distance between sites that recorded shared haplotypes was 10.9 km between MTB2 and MTB6. Sites MTB5, MTB6 and MTB14 all produced unique haplotypes for each individual collected from that site. Individuals from site MTB4 were present in the widest range of MTB haplotypes; five of these seven haplotypes were unique to that site, producing the largest number of unique haplotypes in the Mt Buffalo region.

### 3.3 SNP broadscale analyses

**FIGURE 4:**
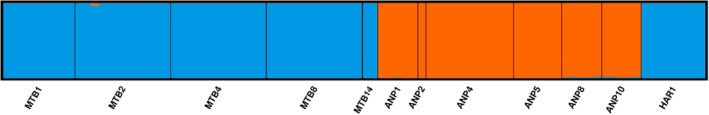
Full dataset Individual proportions of Ancestry Coefficients of *Austrochloritis* spp. populations identified into clusters. Clusters are denoted by colour.

**FIGURE 5:**
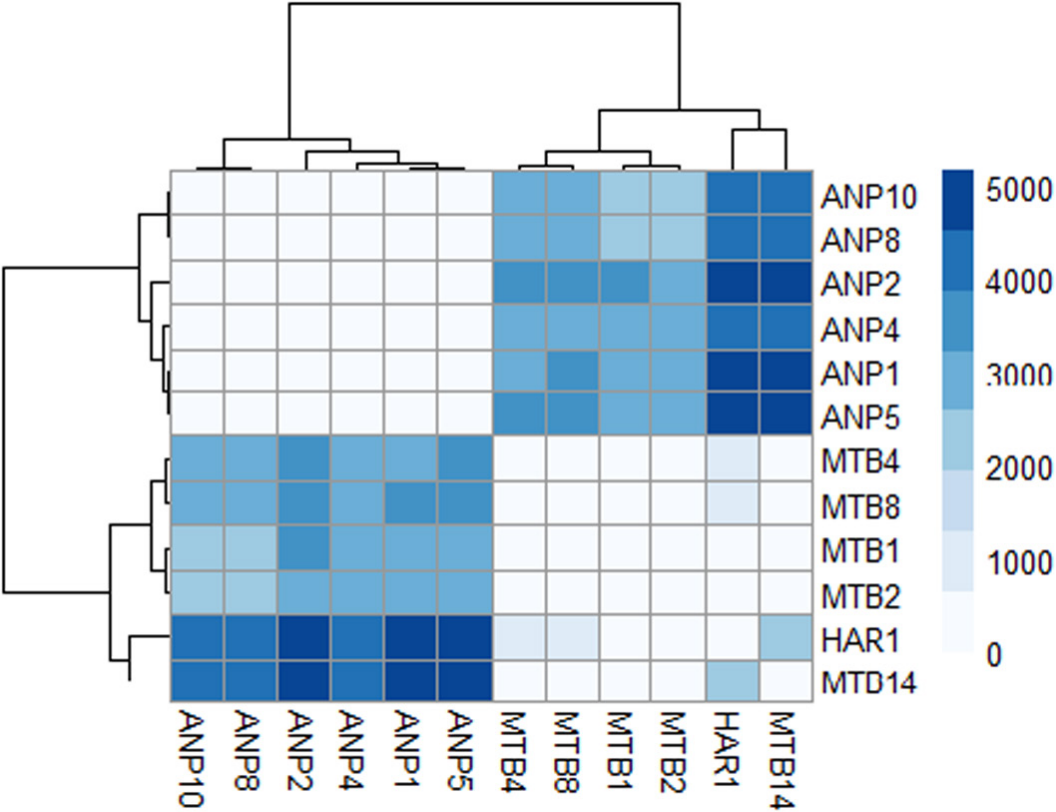
Heatmap reflecting the phylogenetic frequencies reflected between populations, darker tiles represent highly differentiated populations, while light tiles represent closely related populations. Produced from the pheatmap package in R version 4.1.1.

All analyses of the full SNP dataset suggest notable differentiation between regions. The PCA suggests distinct clusters splitting Kiewa and Ovens into separate populations, and Mt Buffalo into multiple populations (Figure S3); the DAPC analysis suggests 6 populations with Kiewa and Ovens forming separate populations, and Mt Buffalo split into four populations (Figure S4, Table 2), while the Structure plot only identifies two, separating the Kiewa sites from the Mt Buffalo and Ovens sites (Figure 4, Table 2); however, further Structure analyses within each of these groups demonstrated the presence of additional clusters. The fixed‐difference analysis (Figure 5, Table 2, Table S4) found that samples from Kiewa produced over 5000 fixed differences with those from both MTB and Ovens, and demonstrated lower differentiation between the Mt Buffalo and Ovens samples, with ~200 to ~2000 fixed differences between these snails, while there were minimal fixed differences present between sites within each region. The differences between regions are also supported by the very high pairwise *F*_ST_ values produced (Table S5), with values ranging from 0.59 to 0.89 between Kiewa and MTB sites, 0.85 to 0.90 between Ovens and Kiewa sites and 0.39 to 0.72 between Ovens and MTB sites. Unfortunately, samples from MTB12 could not be used for any SNP analyses, so a comparison between mtDNA and SNP analysis results for MTB haplogroup 2 is not possible.

The BFD* results supported the species status of all clusters identified by the Structure analysis, including the minor differences shown between the Kiewa populations (see below). Support for this model was significantly greater than any other model of speciation tested (Table S6), although it must be noted that we could not test the model of a single species due to a lack of data from outgroup taxa. This support for a high number of species splits is contrary to much of the evidence from mtDNA, fixed difference analysis and even the initial Structure results and may be due to the tendency for many coalescent‐based species delimitation approaches to over‐split species.

### 3.4 SNP fine‐scale analyses

Based on the full SNP dataset, genetic diversity measurements (Table S7) revealed that within MTB populations, heterozygosity (Ho = 0.13–0.15), inbreeding coefficients (Fis = 0.03–0.23) and allelic richness (AR = 1.16–1.19) were higher than both Ovens (Ho = 0.06, Hs = 0.07, Fis = 0.08, and AR = 1.06) and Kiewa populations (Ho = 0.02–0.05, Hs = 0.03–0.06, Fis = 0.003–0.06, and AR = 1.02–1.06). There is negligible difference between observed (Ho) and expected heterozygosity (He) for the full and Kiewa datasets; however, the MTB dataset produced Ho values (0.185–0.210) in each site that were noticeably lower than the He values (0.231–0.253) produced for each site.

**FIGURE 6:**
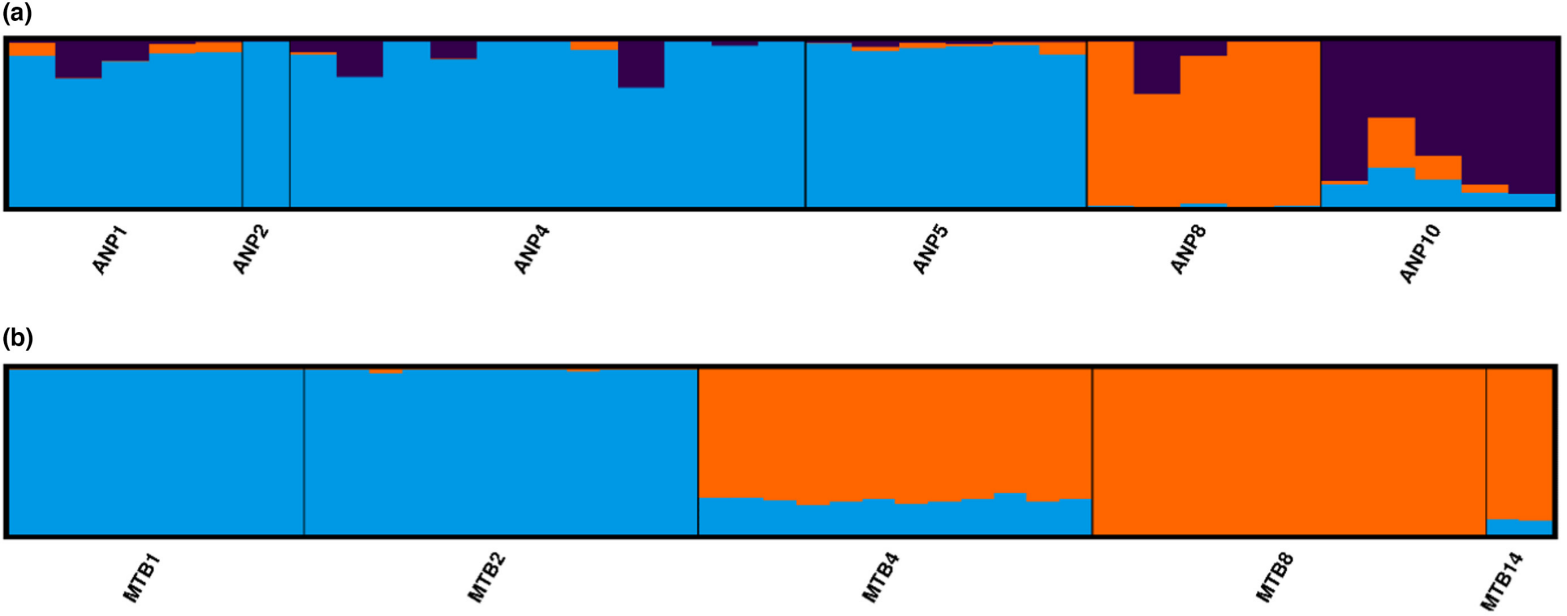
Individual proportions of Ancestry Coefficients of *Austrochloritis* spp. populations identified into clusters. Clusters are denoted by colour. (a) Just Kiewa samples. (b) Just MTB samples.

The PCA and Structure plots produced for both the MTB and Kiewa datasets (Figure S5, Figure 6) show evidence for population structure existing within both regions. Genetic distances between the MTB sites are more pronounced (Figure S5a, Figure 6b), with sites MTB1 and MTB2 forming one cluster while the remaining three sites (MTB4, MTB8 and MTB14) form a second cluster. This is moderately supported by the *F*_ST_ values produced between these sites (Table S5), with sites MTB1 and MTB2 sharing a lower *F*_ST_ value with one another (0.13) than with the remaining sites, MTB4 (0.28 and 0.23) and MTB8 (0.37 and 0.33). The almost identical admixture shared between MTB1 and MTB2 suggests the presence of a single population on the southern slopes of Mt Buffalo along the Buckland River Valley. The differentiation between sample sites is further supported by isolation‐by‐distance plots (Figure S6b), showing that that differences between Mt Buffalo samples can be explained by distance between collection sites, with as little as 10 km preventing between‐site gene flow. When the Ovens site was included in the MTB dataset Structure analysis (Figure S7), it formed a separate cluster, and did not appear as genetically similar to samples from MTB1 or MTB2 as was seen in the mtDNA data.

Migration rates produced between MTB sites (Table 3a) displayed evidence of migration occurring from MTB2 (345 m Above Sea Level (ASL)) to MTB1 (319 m ASL) (m = 0.12, 95% CI: 0.046–0.194), and from MTB14 (911 m ASL) to MTB4 (473 m ASL) (m = 0.17, 95% CI: 0.06–0.28), but there was little evidence of migration occurring between the two clusters of MTB sites with the majority of migration rates between them not significantly different from 0. However, there was evidence of migration from MTB4 (473 m ASL) to MTB2 (345 m ASL) (m = 0.12, 95% CI: 0.046–0.19), but there was no significant evidence for migration occurring between MTB4 and the geographically closer site MTB1. Every migration between Mt Buffalo sites was from a higher elevation to a lower elevation.

**TABLE 3:** Asymmetric migration rate between pairs of sites for *Austrochloritis kosciuszkoensis* estimated from BAYEASS.

FROM	INTO				
(a)	MTB1	MTB2	MTB4	MTB8	MTB14
MTB1	0.86 (0.084)	0.024 (0.043)	0.024 (0.044)	0.072 (0.069)	0.024 (0.044)
MTB2	0.12 (0.074)	0.80 (0.076)	0.039 (0.049)	0.020 (0.036)	0.020 (0.036)
MTB4	0.020 (0.036)	0.12 (0.074)	0.82 (0.077)	0.020 (0.036)	0.020 (0.036)
MTB8	0.021 (0.038)	0.042 (0.053)	0.083 (0.069)	0.83 (0.079)	0.021 (0.038)
MTB14	0.042 (0.072)	0.042 (0.072)	0.17 (0.11)	0.042 (0.072)	0.71 (0.072)

FROM	INTO					
(b)	ANP1	ANP2	ANP4	ANP5	ANP8	ANP10
ANP1	0.70 (0.054)	0.030 (0.054)	0.18 (0.094)	0.030 (0.054)	0.030 (0.054)	0.030 (0.054)
ANP2	0.042 (0.072)	0.71 (0.072)	0.12 (0.10)	0.042 (0.072)	0.042 (0.072)	0.042 (0.073)
ANP4	0.020 (0.036)	0.020 (0.036)	0.86 (0.076)	0.059 (0.058)	0.020 (0.037)	0.020 (0.036)
ANP5	0.031 (0.054)	0.030 (0.054)	0.061 (0.073)	0.82 (0.094)	0.031 (0.054)	0.030 (0.054)
ANP8	0.028 (0.051)	0.028 (0.050)	0.11 (0.085)	0.056 (0.067)	0.75 (0.078)	0.028 (0.050)
ANP10	0.034 (0.060)	0.034 (0.059)	0.13 (0.096)	0.034 (0.059)	0.033 (0.059)	0.73 (0.079)

*Note*: Tables are intended to be read from row to column, so values represent the migration rate of regions in the row headings into regions in column headings. Values in brackets are ±95% confidence intervals. (above) MTB sites, and (below) Kiewa sites.

There is less gene flow evident in the Kiewa samples with three clusters present (Figure 6a, Figure S5b). Sites ANP1, ANP4 and ANP5 (1–2 km apart) are all placed in the same cluster, which is also supported by the *F*_ST_ analysis (Table S5; 0.03–0.05). In contrast, ANP8 and ANP10 both formed separate populations and displayed higher *F*_ST_ values. An Isolation‐by‐distance plot (Figure S6a) displayed similar results to those found from MTB, with distance linked to observed differences between samples.

Migration rates produced between Kiewa sites (Table 3b) displayed evidence of migration occurring from every site into ANP4 except ANP5 (ANP1 (696 m ASL) to ANP4 (762 m ASL) (m = 0.18, 95% CI: 0.086–0.274), ANP2 (1197 m ASL) to ANP4 (m = 0.12, 95% CI: 0.02–0.22), ANP8 (464 m ASL) to ANP4 (m = 0.11, 95% CI: 0.025–0.195), and ANP10 (627 m ASL) to ANP4 (m = 0.13, 95% CI: 0.034–0.226)). Excluding ANP2, every other migration into ANP4 was from a lower elevation. The majority of migration rates between the remaining sites included 0 in the 95% confidence intervals.

## 4 DISCUSSION

Our results show that putative *A. kosciuszkoensis* snails from our two main study regions, from only a fraction of its total distribution, are clearly genetically differentiated from each other and may represent distinct species. At the finer geographic scale, the SNP data revealed evidence of significant population differentiation at some sites within these two regions, demonstrating a significant lack of gene flow over even very small geographic scales. The discovery of very low gene flow and large genetic differences implies that these land snails do not disperse well, suggesting that multiple new species are possible across the range of *A. kosciuszkoensis*.

### 4.1 Broad‐scale population structure and lineage divergence

The degree of divergence found within the small range covered by this study (30–50 km) suggests that the *Austrochloritis* specimens used in this study may not belong to the same species, *A. kosciuszkoensis*. The sites studied cover only a portion of the currently accepted distribution of *A. kosciuszkoensis* (Köhler et al., 2020; Stanisic et al., 2010) and are over 100 km away from the type locality in Kosciuszko National Park. Given the patterns of substantial genetic turn‐over across rather short geographic distances, the taxonomic identity across *A. kosciuszkoensis* is in need of further scrutiny. Moreover, all species delimitation approaches, for both nuclear and mtDNA data, suggest that the large genetic differences detected between the Mt Buffalo and Kiewa regions represent separate species. However, given the continuous debate of how to properly define species (Coyne & Allen Orr, 1998; Mallet, 1995; Wu et al., 2020), populations from these two regions could instead be considered Evolutionary Significant Units (ESU). Regardless of how they are defined, there are undeniably large genetic differences between the Kiewa and Mt Buffalo populations.

However, the species delimitation methods here do not provide a clear answer to the number of taxa present in the sampled regions. While there is agreement that the Kiewa and Mt Buffalo populations are different taxa, many of the delimitation approaches also suggest the Harrietville/Ovens River and Mt Buffalo lineages represent different species, and the most extreme delimitation, generated by the coalescent approach, suggests that every cluster separated by Structure is a different species. These results highlight some of the inherent difficulties of delimiting species genetically (Carstens et al., 2013; Naciri & Linder, 2015; Sukumaran & Knowles, 2017), and provide further evidence that caution is required when using genetic approaches to place species boundaries on taxa that consist of isolated allopatric populations (Smith & Carstens, 2020).

Inferences regarding deep lineage diversification present in this study are further complicated by apparent mismatches between mtDNA and SNP data. For instance, the multiple deep mtDNA lineages present within Mt Buffalo were suggestive of sympatric species; however, the SNP data produced no evidence for a significant divergence among samples from those sites. It is likely then that some of the mitochondrial lineage divergence seen in this study is due to retention of ancestral polymorphisms from a previous divergence event and is not indicative of speciation (Guerrero & Hahn, 2017). Given the degree of mtDNA divergence present, the data suggests that past palaeoclimatic events may have isolated lineages enough to evolve separate mtDNA lineages with subsequent expansion events enabling distinct haplogroups to mix throughout Mt Buffalo. Similar patterns of expanding and contracting ranges have been observed in other invertebrate species (Garrick et al., 2012; Hatley & Murphy, 2016; Pinceel et al., 2005). Ancestral polymorphisms are not uncommon in leading to misinterpretations of mtDNA data in diversity studies by increasing the level of genetic diversity observed between individuals (Cutter & Choi, 2010; Guerrero & Hahn, 2017). Ancestral polymorphisms can also lead to overestimates of species‐level differences (Naciri & Linder, 2015; Petit & Excoffier, 2009), hence the suggestion that multi‐gene approaches to species lists are preferable over single‐gene delimitation efforts. We suggest that confusion surrounding species limits in these snails should be resolved by including a traditional morphological approach, involving inspection of the external morphology and internal anatomy to determine the taxonomic status of these individuals.

In spite of some doubts of taxonomic status, both our findings and the history of cryptic species within the *Austrochloritis* genus (Köhler et al., 2020; Shea et al., 2020) suggest that this group of terrestrial snails consists of multiple narrow range endemic species, as has been demonstrated for other invertebrate species at the studied area, such as glow worms (Baker et al., 2008), springtails (Endo et al., 2015), butterflies (Dunn, 2019) and grass hoppers (Tatarnic et al., 2013; Umbers et al., 2022). In particular, these studies suggest that endemism in the Mt Buffalo region should be investigated further to determine the extent of unique evolutionary diversity present, and the landscape characteristics, which create such unique lineages.

### 4.2 Fine‐scale structure

The strong signal of allopatric divergence across the deeper lineages of the dataset is supported by the presence of highly structured populations. The largest distance between sites with evidence of ongoing gene flow was as little as 3.34 km, and still, some sites separated by less than this distance were genetically unique. While population isolation of this scale has been reported previously among poorly‐dispersing species (Hatley & Murphy, 2016; Pinceel et al., 2005), the distance at which these populations are genetically isolated is notable.

Given the low number of snails observed at our collection sites and the rarity of many Australian land snails in general (Butcher & Grove, 2003; Parkyn & Newell, 2013), it is possible that the genetic differences observed between sites are not only driven by low gene flow, but are also a result of multiple local bottleneck events and genetic drift (Barton & Charlesworth, 1984). There is some evidence of this in the Mt Buffalo sites, with lower observed heterozygosity and high Fis suggesting that this region has experienced population bottlenecks, or it could be a product of selfing which has been observed in other snail species (Monsutti & Perrin, 1999; Walters et al., 2022). Events that have driven potential bottlenecks in Mt Buffalo snails do not appear to have impacted the Kiewa snails, and so may be related to the isolated “island” nature of Mt Buffalo itself, meaning that disturbance events such as the frequent large‐scale bushfires that have burnt the entire national park have a disproportionate impact on poorly dispersing terrestrial invertebrates.

Highly structured populations occur at sites falling within the same tract of continuous forest, suggesting that naturally occurring landscape features such as catchments may be critical for shaping the genetic structure in land snails. This is especially the case for the large species‐level divergences separating the snails from the Kiewa populations to those from Harrietville and Mt Buffalo, both of which are situated in the Ovens River Drainage. Catchment‐based diversity between populations of short‐ranged invertebrates, even on a microgeographic scale, is well documented in species like flatworms (Garrick et al., 2012), springtails (Garrick et al., 2007, 2012) and amphipods (Hatley & Murphy, 2016). While at the larger scale catchments appear to be important for lineage divergence, there is less evidence that they play a role at the population level, although we acknowledge that the difficulty of collecting terrestrial snails means that this is hard to test thoroughly. Factors driving connectivity and isolation appear to be multifaceted or are simply due to geographic distance. For example, the Kiewa populations are separated according to altitude in a continuous forested landscape: sites at similar elevations exhibit genetically similar populations, with migration between sites occurring from lower elevations into higher elevations. Populations do not appear to be inherently differentiated by sub‐catchments, as sites ANP8 and ANP10 fall within the same Kiewa River East Brach sub‐catchment but are genetically distinct, while sites ANP1, ANP2, ANP4 and ANP5 are clustered together despite all belonging to separate sub‐catchments. In contrast, elevation appears to be less important among Mt Buffalo sites. Here, the sites that display greater evidence of gene flow are located in different sub‐catchments across different elevations, with migration between sites occurring from a higher elevation to a lower elevation. This pattern suggests that factors driving genetic isolation in one geographic area may not be significant elsewhere. Anthropogenic land use history (e.g. land clearing for homes and farmland or forestry) may have played some role in contemporary population isolation along the same catchment (Ng et al., 2018), but this needs further investigation. A larger study is required to determine the role of factors such as altitude, land use, catchments and past disturbances in order to identify the limit of less mobile SRE species' dispersal abilities.

## 5 CONCLUSIONS

The *Austrochloritis* populations included in this study demonstrate that this species exhibits poor dispersal ability, even more so than originally predicted, to the point where species‐level differences are apparent on a small scale and taxonomic status should be clarified. Given an aim of this study was to use *A. kosciuszkoensis* as a proxy for other, even more poorly known and less mobile taxa, the results presented here provide a level of concern for other, especially smaller SRE species, which are likely to exhibit even more distinct isolated populations. These taxa are at risk due to increasing disturbance events, both natural and anthropogenic (Brooker et al., 2007; Köhler et al., 2020). Clearly, more work is required to understand our terrestrial invertebrate fauna. A large number of groups are likely to exhibit similar dispersal limitations and, as such, similar highly structured populations and lineage diversity, which should be conserved.

Mt Buffalo National Park represents a unique opportunity for observation of dispersal‐poor SRE invertebrate species, due to exhibiting a unique, range of “island” habitats in an alpine region that likely contain short‐ranged invertebrate species and populations with unique evolutionary histories that warrant inquiry. The discovery of mito‐nuclear discordance between individuals within these species is worthy of further inquiry because it adds to the complexity of species delineation in this system.

## AUTHOR CONTRIBUTIONS

**Lachlan J. Gretgrix:** Data curation (lead); formal analysis (lead); investigation (lead); visualization (lead); writing – original draft (lead); writing – review and editing (equal). **Orsi Decker:** Conceptualization (equal); data curation (supporting); investigation (supporting); project administration (equal); resources (equal); supervision (equal); writing – original draft (supporting); writing – review and editing (equal). **Peter T. Green:** Conceptualization (equal); funding acquisition (lead); project administration (equal); resources (equal); supervision (equal); writing – original draft (supporting); writing – review and editing (equal). **Frank Köhler:** Conceptualization (equal); writing – review and editing (equal). **Adnan Moussalli:** Conceptualization (equal); writing – review and editing (equal). **Nicholas P. Murphy:** Conceptualization (lead); methodology (lead); project administration (equal); resources (equal); supervision (equal); writing – original draft (supporting); writing – review and editing (equal).

## CONFLICT OF INTEREST STATEMENT

The authors declare no conflicts of interest.

## Supporting information


Appendix S1

## Data Availability

Data files are accessible through the following link: https://www.dropbox.com/scl/fo/wh5rypxtvbj861yazafmh/h?rlkey=99nyflu5o4e6cjyw1aqnjaphy&dl=0. Please refer to the README.txt file for instructions for completing analyses. R scripts and data will be permanently archived upon publication on the Dryad data depository.
