# Supplementary material for: Genetic diversity of a short‐ranged endemic terrestrial snail
Source: Ecol Evol. 2023 Nov 28;13(11):e10785. doi: 10.1002/ece3.10785 (PMC10684984; doi:10.1002/ece3.10785)
Supplement: Supplementary file 1 — Appendix S1 [file ECE3-13-e10785-s001.docx]

**APPEN****DIX**

Figure S1. All haplotypes Median-Joining Network of *Austrochloritis* spp. mitochondrial haplotypes. Each circle represents a unique haplotype, circle size is proportionate to the number of individuals carrying the haplotype, black circles represent unsampled haplotypes, lines denote the number of base pair differences between haplotypes, and colours represent the NP/region the samples belong to.

Table S1. mtDNA Haplotype % identity Matrix. Red cells represent the highest percent identity between samples (99.83%) and blue cells represent the lowest percent identity between samples (90.63%). Standard Deviation = 3.22. Calculated in Geneious Prime Vers. 2021.2.2.

|  | **MTB_1** | **MTB_2** | **MTB_3** | **MTB_4** | **MTB_5** | **MTB_6** | **MTB_7** | **MTB_8** | **MTB_9** | **MTB_10** | **MTB_11** | **MTB_12** | **MTB_13** | **MTB_14** | **MTB_15** | **MTB_16** | **MTB_17** | **MTB_18** | **MTB_19** | **MTB_20** | **MTB_21** | **MTB_22** | **MTB_23** | **MTB_24** | **HAR_1** | **HAR_2** | **HAR_3** | **ANP_1** | **ANP_2** | **ANP_3** | **ANP_4** | **ANP_5** | **ANP_6** | **ANP_7** | **ANP_8** | **ANP_9** | **ANP_10** | **ANP_11** | **ANP_12** | **ANP_13** |
| --- | --- | --- | --- | --- | --- | --- | --- | --- | --- | --- | --- | --- | --- | --- | --- | --- | --- | --- | --- | --- | --- | --- | --- | --- | --- | --- | --- | --- | --- | --- | --- | --- | --- | --- | --- | --- | --- | --- | --- | --- |
| **MTB_1** |  | 98.98 | 97.96 | 97.79 | 97.96 | 98.13 | 98.3 | 98.13 | 97.96 | 98.3 | 98.3 | 98.47 | 98.81 | 98.64 | 98.47 | 98.47 | 98.81 | 98.47 | 98.64 | 90.63 | 91.65 | 91.82 | 91.48 | 91.65 | 91.99 | 92.16 | 91.82 | 90.97 | 91.14 | 90.97 | 91.14 | 91.14 | 91.31 | 91.31 | 90.97 | 90.97 | 91.31 | 91.31 | 90.97 | 90.97 |
| **MTB_2** | 98.98 |  | 98.98 | 98.81 | 98.98 | 99.15 | 99.32 | 99.15 | 98.98 | 98.3 | 98.64 | 98.81 | 98.81 | 98.64 | 98.81 | 98.81 | 99.15 | 98.81 | 98.98 | 91.65 | 92.33 | 92.5 | 92.16 | 92.33 | 92.67 | 92.84 | 92.5 | 91.65 | 91.82 | 91.65 | 91.82 | 91.82 | 91.99 | 91.99 | 91.65 | 91.65 | 91.99 | 91.99 | 91.65 | 91.65 |
| **MTB_3** | 97.96 | 98.98 |  | 99.83 | 99.66 | 99.83 | 99.66 | 99.49 | 99.66 | 98.98 | 99.32 | 99.15 | 99.15 | 98.98 | 99.15 | 99.15 | 99.15 | 99.15 | 99.32 | 92.5 | 93.19 | 93.36 | 93.02 | 93.19 | 93.53 | 93.7 | 93.36 | 92.5 | 92.67 | 92.5 | 92.67 | 92.67 | 92.84 | 92.84 | 92.5 | 92.5 | 92.84 | 92.84 | 92.5 | 92.5 |
| **MTB_4** | 97.79 | 98.81 | 99.83 |  | 99.49 | 99.66 | 99.49 | 99.32 | 99.49 | 98.81 | 99.15 | 98.98 | 98.98 | 98.81 | 98.98 | 98.98 | 98.98 | 98.98 | 99.15 | 92.33 | 93.02 | 93.19 | 92.84 | 93.02 | 93.36 | 93.53 | 93.19 | 92.33 | 92.5 | 92.33 | 92.5 | 92.5 | 92.67 | 92.67 | 92.33 | 92.33 | 92.67 | 92.67 | 92.33 | 92.33 |
| **MTB_5** | 97.96 | 98.98 | 99.66 | 99.49 |  | 99.83 | 99.66 | 99.49 | 99.66 | 98.64 | 99.32 | 99.15 | 99.15 | 98.98 | 99.15 | 99.15 | 99.15 | 99.15 | 99.32 | 92.5 | 92.84 | 93.02 | 92.67 | 92.84 | 93.19 | 93.36 | 93.02 | 92.16 | 92.33 | 92.16 | 92.33 | 92.33 | 92.5 | 92.5 | 92.16 | 92.16 | 92.5 | 92.5 | 92.16 | 92.16 |
| **MTB_6** | 98.13 | 99.15 | 99.83 | 99.66 | 99.83 |  | 99.83 | 99.66 | 99.83 | 98.81 | 99.49 | 99.32 | 99.32 | 99.15 | 99.32 | 99.32 | 99.32 | 99.32 | 99.49 | 92.33 | 93.02 | 93.19 | 92.84 | 93.02 | 93.36 | 93.53 | 93.19 | 92.33 | 92.5 | 92.33 | 92.5 | 92.5 | 92.67 | 92.67 | 92.33 | 92.33 | 92.67 | 92.67 | 92.33 | 92.33 |
| **MTB_7** | 98.3 | 99.32 | 99.66 | 99.49 | 99.66 | 99.83 |  | 99.83 | 99.66 | 98.98 | 99.32 | 99.49 | 99.49 | 99.32 | 99.49 | 99.49 | 99.49 | 99.49 | 99.66 | 92.16 | 92.84 | 93.02 | 92.67 | 92.84 | 93.19 | 93.36 | 93.02 | 92.16 | 92.33 | 92.16 | 92.33 | 92.33 | 92.5 | 92.5 | 92.16 | 92.16 | 92.5 | 92.5 | 92.16 | 92.16 |
| **MTB_8** | 98.13 | 99.15 | 99.49 | 99.32 | 99.49 | 99.66 | 99.83 |  | 99.83 | 98.81 | 99.15 | 99.32 | 99.32 | 99.15 | 99.32 | 99.32 | 99.32 | 99.32 | 99.49 | 91.99 | 93.02 | 93.19 | 92.84 | 93.02 | 93.02 | 93.19 | 92.84 | 91.99 | 92.16 | 91.99 | 92.16 | 92.16 | 92.33 | 92.33 | 91.99 | 91.99 | 92.33 | 92.33 | 91.99 | 91.99 |
| **MTB_9** | 97.96 | 98.98 | 99.66 | 99.49 | 99.66 | 99.83 | 99.66 | 99.83 |  | 98.64 | 99.32 | 99.15 | 99.15 | 98.98 | 99.15 | 99.15 | 99.15 | 99.15 | 99.32 | 92.16 | 93.19 | 93.36 | 93.02 | 93.19 | 93.19 | 93.36 | 93.02 | 92.16 | 92.33 | 92.16 | 92.33 | 92.33 | 92.5 | 92.5 | 92.16 | 92.16 | 92.5 | 92.5 | 92.16 | 92.16 |
| **MTB_10** | 98.3 | 98.3 | 98.98 | 98.81 | 98.64 | 98.81 | 98.98 | 98.81 | 98.64 |  | 98.98 | 99.15 | 99.49 | 99.32 | 99.15 | 99.15 | 99.15 | 99.15 | 99.32 | 91.48 | 92.5 | 92.67 | 92.33 | 92.5 | 92.84 | 93.02 | 92.67 | 92.16 | 92.33 | 92.16 | 92.33 | 92.33 | 92.5 | 92.5 | 92.16 | 92.16 | 92.5 | 92.5 | 92.16 | 92.16 |
| **MTB_11** | 98.3 | 98.64 | 99.32 | 99.15 | 99.32 | 99.49 | 99.32 | 99.15 | 99.32 | 98.98 |  | 99.83 | 99.49 | 99.32 | 99.49 | 99.49 | 99.49 | 99.49 | 99.66 | 91.82 | 92.84 | 93.02 | 92.67 | 92.84 | 93.19 | 93.36 | 93.02 | 91.82 | 91.99 | 91.82 | 91.99 | 91.99 | 92.16 | 92.16 | 91.82 | 91.82 | 92.16 | 92.16 | 91.82 | 91.82 |
| **MTB_12** | 98.47 | 98.81 | 99.15 | 98.98 | 99.15 | 99.32 | 99.49 | 99.32 | 99.15 | 99.15 | 99.83 |  | 99.66 | 99.49 | 99.66 | 99.66 | 99.66 | 99.66 | 99.83 | 91.65 | 92.67 | 92.84 | 92.5 | 92.67 | 93.02 | 93.19 | 92.84 | 91.65 | 91.82 | 91.65 | 91.82 | 91.82 | 91.99 | 91.99 | 91.65 | 91.65 | 91.99 | 91.99 | 91.65 | 91.65 |
| **MTB_13** | 98.81 | 98.81 | 99.15 | 98.98 | 99.15 | 99.32 | 99.49 | 99.32 | 99.15 | 99.49 | 99.49 | 99.66 |  | 99.83 | 99.66 | 99.66 | 99.66 | 99.66 | 99.83 | 91.65 | 92.67 | 92.84 | 92.5 | 92.67 | 93.02 | 93.19 | 92.84 | 91.99 | 92.16 | 91.99 | 92.16 | 92.16 | 92.33 | 92.33 | 91.99 | 91.99 | 92.33 | 92.33 | 91.99 | 91.99 |
| **MTB_14** | 98.64 | 98.64 | 98.98 | 98.81 | 98.98 | 99.15 | 99.32 | 99.15 | 98.98 | 99.32 | 99.32 | 99.49 | 99.83 |  | 99.49 | 99.49 | 99.49 | 99.49 | 99.66 | 91.48 | 92.5 | 92.67 | 92.33 | 92.5 | 92.84 | 93.02 | 92.67 | 91.82 | 91.99 | 91.82 | 91.99 | 91.99 | 92.16 | 92.16 | 91.82 | 91.82 | 92.16 | 92.16 | 91.82 | 91.82 |
| **MTB_15** | 98.47 | 98.81 | 99.15 | 98.98 | 99.15 | 99.32 | 99.49 | 99.32 | 99.15 | 99.15 | 99.49 | 99.66 | 99.66 | 99.49 |  | 99.66 | 99.66 | 99.66 | 99.83 | 91.65 | 92.67 | 92.84 | 92.5 | 92.67 | 93.02 | 93.19 | 92.84 | 91.65 | 91.82 | 91.65 | 91.82 | 91.82 | 91.99 | 91.99 | 91.65 | 91.65 | 91.99 | 91.99 | 91.65 | 91.65 |
| **MTB_16** | 98.47 | 98.81 | 99.15 | 98.98 | 99.15 | 99.32 | 99.49 | 99.32 | 99.15 | 99.15 | 99.49 | 99.66 | 99.66 | 99.49 | 99.66 |  | 99.66 | 99.66 | 99.83 | 91.65 | 92.67 | 92.84 | 92.5 | 92.67 | 93.02 | 93.19 | 92.84 | 91.99 | 92.16 | 91.99 | 92.16 | 92.16 | 92.33 | 92.33 | 91.99 | 91.99 | 92.33 | 92.33 | 91.99 | 91.99 |
| **MTB_17** | 98.81 | 99.15 | 99.15 | 98.98 | 99.15 | 99.32 | 99.49 | 99.32 | 99.15 | 99.15 | 99.49 | 99.66 | 99.66 | 99.49 | 99.66 | 99.66 |  | 99.66 | 99.83 | 91.65 | 92.67 | 92.84 | 92.5 | 92.67 | 93.02 | 93.19 | 92.84 | 91.65 | 91.82 | 91.65 | 91.82 | 91.82 | 91.99 | 91.99 | 91.65 | 91.65 | 91.99 | 91.99 | 91.65 | 91.65 |
| **MTB_18** | 98.47 | 98.81 | 99.15 | 98.98 | 99.15 | 99.32 | 99.49 | 99.32 | 99.15 | 99.15 | 99.49 | 99.66 | 99.66 | 99.49 | 99.66 | 99.66 | 99.66 |  | 99.83 | 91.99 | 92.67 | 92.84 | 92.5 | 92.67 | 93.02 | 93.19 | 92.84 | 91.65 | 91.82 | 91.65 | 91.82 | 91.82 | 91.99 | 91.99 | 91.65 | 91.65 | 91.99 | 91.99 | 91.65 | 91.65 |
| **MTB_19** | 98.64 | 98.98 | 99.32 | 99.15 | 99.32 | 99.49 | 99.66 | 99.49 | 99.32 | 99.32 | 99.66 | 99.83 | 99.83 | 99.66 | 99.83 | 99.83 | 99.83 | 99.83 |  | 91.82 | 92.84 | 93.02 | 92.67 | 92.84 | 93.19 | 93.36 | 93.02 | 91.82 | 91.99 | 91.82 | 91.99 | 91.99 | 92.16 | 92.16 | 91.82 | 91.82 | 92.16 | 92.16 | 91.82 | 91.82 |
| **MTB_20** | 90.63 | 91.65 | 92.5 | 92.33 | 92.5 | 92.33 | 92.16 | 91.99 | 92.16 | 91.48 | 91.82 | 91.65 | 91.65 | 91.48 | 91.65 | 91.65 | 91.65 | 91.99 | 91.82 |  | 93.36 | 92.84 | 93.19 | 93.02 | 93.36 | 93.36 | 93.19 | 93.7 | 93.87 | 93.36 | 93.53 | 93.87 | 94.04 | 94.04 | 93.7 | 93.7 | 93.7 | 93.7 | 94.04 | 93.7 |
| **MTB_21** | 91.65 | 92.33 | 93.19 | 93.02 | 92.84 | 93.02 | 92.84 | 93.02 | 93.19 | 92.5 | 92.84 | 92.67 | 92.67 | 92.5 | 92.67 | 92.67 | 92.67 | 92.67 | 92.84 | 93.36 |  | 99.49 | 99.83 | 99.66 | 99.32 | 99.15 | 99.15 | 95.23 | 95.4 | 95.23 | 95.4 | 95.74 | 95.91 | 95.91 | 95.23 | 95.23 | 95.23 | 95.23 | 95.23 | 95.23 |
| **MTB_22** | 91.82 | 92.5 | 93.36 | 93.19 | 93.02 | 93.19 | 93.02 | 93.19 | 93.36 | 92.67 | 93.02 | 92.84 | 92.84 | 92.67 | 92.84 | 92.84 | 92.84 | 92.84 | 93.02 | 92.84 | 99.49 |  | 99.66 | 99.83 | 99.15 | 98.98 | 98.98 | 95.06 | 95.23 | 95.4 | 95.57 | 95.57 | 95.4 | 95.74 | 95.06 | 95.06 | 95.06 | 95.06 | 95.06 | 95.06 |
| **MTB_23** | 91.48 | 92.16 | 93.02 | 92.84 | 92.67 | 92.84 | 92.67 | 92.84 | 93.02 | 92.33 | 92.67 | 92.5 | 92.5 | 92.33 | 92.5 | 92.5 | 92.5 | 92.5 | 92.67 | 93.19 | 99.83 | 99.66 |  | 99.83 | 99.15 | 98.98 | 98.98 | 95.4 | 95.57 | 95.4 | 95.57 | 95.91 | 95.74 | 96.08 | 95.4 | 95.4 | 95.4 | 95.4 | 95.4 | 95.4 |
| **MTB_24** | 91.65 | 92.33 | 93.19 | 93.02 | 92.84 | 93.02 | 92.84 | 93.02 | 93.19 | 92.5 | 92.84 | 92.67 | 92.67 | 92.5 | 92.67 | 92.67 | 92.67 | 92.67 | 92.84 | 93.02 | 99.66 | 99.83 | 99.83 |  | 99.32 | 99.15 | 99.15 | 95.23 | 95.4 | 95.57 | 95.74 | 95.74 | 95.57 | 95.91 | 95.23 | 95.23 | 95.23 | 95.23 | 95.23 | 95.23 |
| **HAR_1** | 91.99 | 92.67 | 93.53 | 93.36 | 93.19 | 93.36 | 93.19 | 93.02 | 93.19 | 92.84 | 93.19 | 93.02 | 93.02 | 92.84 | 93.02 | 93.02 | 93.02 | 93.02 | 93.19 | 93.36 | 99.32 | 99.15 | 99.15 | 99.32 |  | 99.83 | 99.49 | 95.23 | 95.4 | 95.57 | 95.74 | 95.74 | 95.91 | 95.91 | 95.23 | 95.23 | 95.23 | 95.23 | 95.23 | 95.23 |
| **HAR_2** | 92.16 | 92.84 | 93.7 | 93.53 | 93.36 | 93.53 | 93.36 | 93.19 | 93.36 | 93.02 | 93.36 | 93.19 | 93.19 | 93.02 | 93.19 | 93.19 | 93.19 | 93.19 | 93.36 | 93.36 | 99.15 | 98.98 | 98.98 | 99.15 | 99.83 |  | 99.32 | 95.06 | 95.23 | 95.4 | 95.57 | 95.57 | 95.74 | 95.74 | 95.06 | 95.06 | 95.06 | 95.06 | 95.06 | 95.06 |
| **HAR_3** | 91.82 | 92.5 | 93.36 | 93.19 | 93.02 | 93.19 | 93.02 | 92.84 | 93.02 | 92.67 | 93.02 | 92.84 | 92.84 | 92.67 | 92.84 | 92.84 | 92.84 | 92.84 | 93.02 | 93.19 | 99.15 | 98.98 | 98.98 | 99.15 | 99.49 | 99.32 |  | 95.4 | 95.57 | 95.74 | 95.91 | 95.91 | 96.08 | 96.08 | 95.4 | 95.4 | 95.4 | 95.4 | 95.4 | 95.4 |
| **ANP_1** | 90.97 | 91.65 | 92.5 | 92.33 | 92.16 | 92.33 | 92.16 | 91.99 | 92.16 | 92.16 | 91.82 | 91.65 | 91.99 | 91.82 | 91.65 | 91.99 | 91.65 | 91.65 | 91.82 | 93.7 | 95.23 | 95.06 | 95.4 | 95.23 | 95.23 | 95.06 | 95.4 |  | 99.83 | 99.32 | 99.15 | 99.49 | 99.32 | 99.32 | 99.66 | 99.66 | 99.66 | 99.66 | 99.66 | 99.66 |
| **ANP_2** | 91.14 | 91.82 | 92.67 | 92.5 | 92.33 | 92.5 | 92.33 | 92.16 | 92.33 | 92.33 | 91.99 | 91.82 | 92.16 | 91.99 | 91.82 | 92.16 | 91.82 | 91.82 | 91.99 | 93.87 | 95.4 | 95.23 | 95.57 | 95.4 | 95.4 | 95.23 | 95.57 | 99.83 |  | 99.49 | 99.32 | 99.66 | 99.49 | 99.49 | 99.83 | 99.83 | 99.83 | 99.83 | 99.83 | 99.83 |
| **ANP_3** | 90.97 | 91.65 | 92.5 | 92.33 | 92.16 | 92.33 | 92.16 | 91.99 | 92.16 | 92.16 | 91.82 | 91.65 | 91.99 | 91.82 | 91.65 | 91.99 | 91.65 | 91.65 | 91.82 | 93.36 | 95.23 | 95.4 | 95.4 | 95.57 | 95.57 | 95.4 | 95.74 | 99.32 | 99.49 |  | 99.83 | 99.49 | 99.32 | 99.32 | 99.32 | 99.32 | 99.32 | 99.32 | 99.32 | 99.32 |
| **ANP_4** | 91.14 | 91.82 | 92.67 | 92.5 | 92.33 | 92.5 | 92.33 | 92.16 | 92.33 | 92.33 | 91.99 | 91.82 | 92.16 | 91.99 | 91.82 | 92.16 | 91.82 | 91.82 | 91.99 | 93.53 | 95.4 | 95.57 | 95.57 | 95.74 | 95.74 | 95.57 | 95.91 | 99.15 | 99.32 | 99.83 |  | 99.32 | 99.15 | 99.15 | 99.15 | 99.15 | 99.15 | 99.15 | 99.15 | 99.15 |
| **ANP_5** | 91.14 | 91.82 | 92.67 | 92.5 | 92.33 | 92.5 | 92.33 | 92.16 | 92.33 | 92.33 | 91.99 | 91.82 | 92.16 | 91.99 | 91.82 | 92.16 | 91.82 | 91.82 | 91.99 | 93.87 | 95.74 | 95.57 | 95.91 | 95.74 | 95.74 | 95.57 | 95.91 | 99.49 | 99.66 | 99.49 | 99.32 |  | 99.83 | 99.83 | 99.49 | 99.49 | 99.49 | 99.49 | 99.49 | 99.49 |
| **ANP_6** | 91.31 | 91.99 | 92.84 | 92.67 | 92.5 | 92.67 | 92.5 | 92.33 | 92.5 | 92.5 | 92.16 | 91.99 | 92.33 | 92.16 | 91.99 | 92.33 | 91.99 | 91.99 | 92.16 | 94.04 | 95.91 | 95.4 | 95.74 | 95.57 | 95.91 | 95.74 | 96.08 | 99.32 | 99.49 | 99.32 | 99.15 | 99.83 |  | 99.66 | 99.32 | 99.32 | 99.32 | 99.32 | 99.32 | 99.32 |
| **ANP_7** | 91.31 | 91.99 | 92.84 | 92.67 | 92.5 | 92.67 | 92.5 | 92.33 | 92.5 | 92.5 | 92.16 | 91.99 | 92.33 | 92.16 | 91.99 | 92.33 | 91.99 | 91.99 | 92.16 | 94.04 | 95.91 | 95.74 | 96.08 | 95.91 | 95.91 | 95.74 | 96.08 | 99.32 | 99.49 | 99.32 | 99.15 | 99.83 | 99.66 |  | 99.32 | 99.32 | 99.32 | 99.32 | 99.32 | 99.32 |
| **ANP_8** | 90.97 | 91.65 | 92.5 | 92.33 | 92.16 | 92.33 | 92.16 | 91.99 | 92.16 | 92.16 | 91.82 | 91.65 | 91.99 | 91.82 | 91.65 | 91.99 | 91.65 | 91.65 | 91.82 | 93.7 | 95.23 | 95.06 | 95.4 | 95.23 | 95.23 | 95.06 | 95.4 | 99.66 | 99.83 | 99.32 | 99.15 | 99.49 | 99.32 | 99.32 |  | 99.66 | 99.66 | 99.66 | 99.66 | 99.66 |
| **ANP_9** | 90.97 | 91.65 | 92.5 | 92.33 | 92.16 | 92.33 | 92.16 | 91.99 | 92.16 | 92.16 | 91.82 | 91.65 | 91.99 | 91.82 | 91.65 | 91.99 | 91.65 | 91.65 | 91.82 | 93.7 | 95.23 | 95.06 | 95.4 | 95.23 | 95.23 | 95.06 | 95.4 | 99.66 | 99.83 | 99.32 | 99.15 | 99.49 | 99.32 | 99.32 | 99.66 |  | 99.66 | 99.66 | 99.66 | 99.66 |
| **ANP_10** | 91.31 | 91.99 | 92.84 | 92.67 | 92.5 | 92.67 | 92.5 | 92.33 | 92.5 | 92.5 | 92.16 | 91.99 | 92.33 | 92.16 | 91.99 | 92.33 | 91.99 | 91.99 | 92.16 | 93.7 | 95.23 | 95.06 | 95.4 | 95.23 | 95.23 | 95.06 | 95.4 | 99.66 | 99.83 | 99.32 | 99.15 | 99.49 | 99.32 | 99.32 | 99.66 | 99.66 |  | 99.66 | 99.66 | 99.66 |
| **ANP_11** | 91.31 | 91.99 | 92.84 | 92.67 | 92.5 | 92.67 | 92.5 | 92.33 | 92.5 | 92.5 | 92.16 | 91.99 | 92.33 | 92.16 | 91.99 | 92.33 | 91.99 | 91.99 | 92.16 | 93.7 | 95.23 | 95.06 | 95.4 | 95.23 | 95.23 | 95.06 | 95.4 | 99.66 | 99.83 | 99.32 | 99.15 | 99.49 | 99.32 | 99.32 | 99.66 | 99.66 | 99.66 |  | 99.66 | 99.66 |
| **ANP_12** | 90.97 | 91.65 | 92.5 | 92.33 | 92.16 | 92.33 | 92.16 | 91.99 | 92.16 | 92.16 | 91.82 | 91.65 | 91.99 | 91.82 | 91.65 | 91.99 | 91.65 | 91.65 | 91.82 | 94.04 | 95.23 | 95.06 | 95.4 | 95.23 | 95.23 | 95.06 | 95.4 | 99.66 | 99.83 | 99.32 | 99.15 | 99.49 | 99.32 | 99.32 | 99.66 | 99.66 | 99.66 | 99.66 |  | 99.66 |
| **ANP_13** | 90.97 | 91.65 | 92.5 | 92.33 | 92.16 | 92.33 | 92.16 | 91.99 | 92.16 | 92.16 | 91.82 | 91.65 | 91.99 | 91.82 | 91.65 | 91.99 | 91.65 | 91.65 | 91.82 | 93.7 | 95.23 | 95.06 | 95.4 | 95.23 | 95.23 | 95.06 | 95.4 | 99.66 | 99.83 | 99.32 | 99.15 | 99.49 | 99.32 | 99.32 | 99.66 | 99.66 | 99.66 | 99.66 | 99.66 |  |

Table S2. mtDNA haplotype uncorrected p-distance matrix. Red cells represent the highest 10% of p-distance values between samples and blue cells represent the lowest 10% of p-distance values between samples. Calculated in ABGD.

|  | **MTB_1** | **MTB_2** | **MTB_3** | **MTB_4** | **MTB_5** | **MTB_6** | **MTB_7** | **MTB_8** | **MTB_9** | **MTB_10** | **MTB_11** | **MTB_12** | **MTB_13** | **MTB_14** | **MTB_15** | **MTB_16** | **MTB_17** | **MTB_18** | **MTB_19** | **MTB_20** | **MTB_21** | **MTB_22** | **MTB_23** | **MTB_24** | **HAR_1** | **HAR_2** | **HAR_3** | **ANP_1** | **ANP_2** | **ANP_3** | **ANP_4** | **ANP_5** | **ANP_6** | **ANP_7** | **ANP_8** | **ANP_9** | **ANP_10** | **ANP_11** | **ANP_12** | **ANP_13** |
| --- | --- | --- | --- | --- | --- | --- | --- | --- | --- | --- | --- | --- | --- | --- | --- | --- | --- | --- | --- | --- | --- | --- | --- | --- | --- | --- | --- | --- | --- | --- | --- | --- | --- | --- | --- | --- | --- | --- | --- | --- |
| **MTB_1** |  | 0.010 | 0.021 | 0.022 | 0.021 | 0.019 | 0.017 | 0.019 | 0.021 | 0.017 | 0.017 | 0.015 | 0.012 | 0.014 | 0.015 | 0.015 | 0.012 | 0.015 | 0.014 | 0.100 | 0.088 | 0.087 | 0.090 | 0.088 | 0.085 | 0.083 | 0.087 | 0.096 | 0.094 | 0.096 | 0.094 | 0.094 | 0.092 | 0.092 | 0.096 | 0.096 | 0.092 | 0.092 | 0.096 | 0.096 |
| **MTB_2** | 0.010 |  | 0.010 | 0.012 | 0.010 | 0.009 | 0.007 | 0.009 | 0.010 | 0.017 | 0.014 | 0.012 | 0.012 | 0.014 | 0.012 | 0.012 | 0.009 | 0.012 | 0.010 | 0.088 | 0.081 | 0.079 | 0.083 | 0.081 | 0.077 | 0.075 | 0.079 | 0.088 | 0.087 | 0.088 | 0.087 | 0.087 | 0.085 | 0.085 | 0.088 | 0.088 | 0.085 | 0.085 | 0.088 | 0.088 |
| **MTB_3** | 0.021 | 0.010 |  | 0.002 | 0.003 | 0.002 | 0.003 | 0.005 | 0.003 | 0.010 | 0.007 | 0.009 | 0.009 | 0.010 | 0.009 | 0.009 | 0.009 | 0.009 | 0.007 | 0.079 | 0.071 | 0.070 | 0.073 | 0.071 | 0.068 | 0.066 | 0.070 | 0.079 | 0.077 | 0.079 | 0.077 | 0.077 | 0.075 | 0.075 | 0.079 | 0.079 | 0.075 | 0.075 | 0.079 | 0.079 |
| **MTB_4** | 0.022 | 0.012 | 0.002 |  | 0.005 | 0.003 | 0.005 | 0.007 | 0.005 | 0.012 | 0.009 | 0.010 | 0.010 | 0.012 | 0.010 | 0.010 | 0.010 | 0.010 | 0.009 | 0.081 | 0.073 | 0.071 | 0.075 | 0.073 | 0.070 | 0.068 | 0.071 | 0.081 | 0.079 | 0.081 | 0.079 | 0.079 | 0.077 | 0.077 | 0.081 | 0.081 | 0.077 | 0.077 | 0.081 | 0.081 |
| **MTB_5** | 0.021 | 0.010 | 0.003 | 0.005 |  | 0.002 | 0.003 | 0.005 | 0.003 | 0.014 | 0.007 | 0.009 | 0.009 | 0.010 | 0.009 | 0.009 | 0.009 | 0.009 | 0.007 | 0.079 | 0.075 | 0.073 | 0.077 | 0.075 | 0.071 | 0.070 | 0.073 | 0.083 | 0.081 | 0.083 | 0.081 | 0.081 | 0.079 | 0.079 | 0.083 | 0.083 | 0.079 | 0.079 | 0.083 | 0.083 |
| **MTB_6** | 0.019 | 0.009 | 0.002 | 0.003 | 0.002 |  | 0.002 | 0.003 | 0.002 | 0.012 | 0.005 | 0.007 | 0.007 | 0.009 | 0.007 | 0.007 | 0.007 | 0.007 | 0.005 | 0.081 | 0.073 | 0.071 | 0.075 | 0.073 | 0.070 | 0.068 | 0.071 | 0.081 | 0.079 | 0.081 | 0.079 | 0.079 | 0.077 | 0.077 | 0.081 | 0.081 | 0.077 | 0.077 | 0.081 | 0.081 |
| **MTB_7** | 0.017 | 0.007 | 0.003 | 0.005 | 0.003 | 0.002 |  | 0.002 | 0.003 | 0.010 | 0.007 | 0.005 | 0.005 | 0.007 | 0.005 | 0.005 | 0.005 | 0.005 | 0.003 | 0.083 | 0.075 | 0.073 | 0.077 | 0.075 | 0.071 | 0.070 | 0.073 | 0.083 | 0.081 | 0.083 | 0.081 | 0.081 | 0.079 | 0.079 | 0.083 | 0.083 | 0.079 | 0.079 | 0.083 | 0.083 |
| **MTB_8** | 0.019 | 0.009 | 0.005 | 0.007 | 0.005 | 0.003 | 0.002 |  | 0.002 | 0.012 | 0.009 | 0.007 | 0.007 | 0.009 | 0.007 | 0.007 | 0.007 | 0.007 | 0.005 | 0.085 | 0.073 | 0.071 | 0.075 | 0.073 | 0.073 | 0.071 | 0.075 | 0.085 | 0.083 | 0.085 | 0.083 | 0.083 | 0.081 | 0.081 | 0.085 | 0.085 | 0.081 | 0.081 | 0.085 | 0.085 |
| **MTB_9** | 0.021 | 0.010 | 0.003 | 0.005 | 0.003 | 0.002 | 0.003 | 0.002 |  | 0.014 | 0.007 | 0.009 | 0.009 | 0.010 | 0.009 | 0.009 | 0.009 | 0.009 | 0.007 | 0.083 | 0.071 | 0.070 | 0.073 | 0.071 | 0.071 | 0.070 | 0.073 | 0.083 | 0.081 | 0.083 | 0.081 | 0.081 | 0.079 | 0.079 | 0.083 | 0.083 | 0.079 | 0.079 | 0.083 | 0.083 |
| **MTB_10** | 0.017 | 0.017 | 0.010 | 0.012 | 0.014 | 0.012 | 0.010 | 0.012 | 0.014 |  | 0.010 | 0.009 | 0.005 | 0.007 | 0.009 | 0.009 | 0.009 | 0.009 | 0.007 | 0.090 | 0.079 | 0.077 | 0.081 | 0.079 | 0.075 | 0.073 | 0.077 | 0.083 | 0.081 | 0.083 | 0.081 | 0.081 | 0.079 | 0.079 | 0.083 | 0.083 | 0.079 | 0.079 | 0.083 | 0.083 |
| **MTB_11** | 0.017 | 0.014 | 0.007 | 0.009 | 0.007 | 0.005 | 0.007 | 0.009 | 0.007 | 0.010 |  | 0.002 | 0.005 | 0.007 | 0.005 | 0.005 | 0.005 | 0.005 | 0.003 | 0.087 | 0.075 | 0.073 | 0.077 | 0.075 | 0.071 | 0.070 | 0.073 | 0.087 | 0.085 | 0.087 | 0.085 | 0.085 | 0.083 | 0.083 | 0.087 | 0.087 | 0.083 | 0.083 | 0.087 | 0.087 |
| **MTB_12** | 0.015 | 0.012 | 0.009 | 0.010 | 0.009 | 0.007 | 0.005 | 0.007 | 0.009 | 0.009 | 0.002 |  | 0.003 | 0.005 | 0.003 | 0.003 | 0.003 | 0.003 | 0.002 | 0.088 | 0.077 | 0.075 | 0.079 | 0.077 | 0.073 | 0.071 | 0.075 | 0.088 | 0.087 | 0.088 | 0.087 | 0.087 | 0.085 | 0.085 | 0.088 | 0.088 | 0.085 | 0.085 | 0.088 | 0.088 |
| **MTB_13** | 0.012 | 0.012 | 0.009 | 0.010 | 0.009 | 0.007 | 0.005 | 0.007 | 0.009 | 0.005 | 0.005 | 0.003 |  | 0.002 | 0.003 | 0.003 | 0.003 | 0.003 | 0.002 | 0.088 | 0.077 | 0.075 | 0.079 | 0.077 | 0.073 | 0.071 | 0.075 | 0.085 | 0.083 | 0.085 | 0.083 | 0.083 | 0.081 | 0.081 | 0.085 | 0.085 | 0.081 | 0.081 | 0.085 | 0.085 |
| **MTB_14** | 0.014 | 0.014 | 0.010 | 0.012 | 0.010 | 0.009 | 0.007 | 0.009 | 0.010 | 0.007 | 0.007 | 0.005 | 0.002 |  | 0.005 | 0.005 | 0.005 | 0.005 | 0.003 | 0.090 | 0.079 | 0.077 | 0.081 | 0.079 | 0.075 | 0.073 | 0.077 | 0.087 | 0.085 | 0.087 | 0.085 | 0.085 | 0.083 | 0.083 | 0.087 | 0.087 | 0.083 | 0.083 | 0.087 | 0.087 |
| **MTB_15** | 0.015 | 0.012 | 0.009 | 0.010 | 0.009 | 0.007 | 0.005 | 0.007 | 0.009 | 0.009 | 0.005 | 0.003 | 0.003 | 0.005 |  | 0.003 | 0.003 | 0.003 | 0.002 | 0.088 | 0.077 | 0.075 | 0.079 | 0.077 | 0.073 | 0.071 | 0.075 | 0.088 | 0.087 | 0.088 | 0.087 | 0.087 | 0.085 | 0.085 | 0.088 | 0.088 | 0.085 | 0.085 | 0.088 | 0.088 |
| **MTB_16** | 0.015 | 0.012 | 0.009 | 0.010 | 0.009 | 0.007 | 0.005 | 0.007 | 0.009 | 0.009 | 0.005 | 0.003 | 0.003 | 0.005 | 0.003 |  | 0.003 | 0.003 | 0.002 | 0.088 | 0.077 | 0.075 | 0.079 | 0.077 | 0.073 | 0.071 | 0.075 | 0.085 | 0.083 | 0.085 | 0.083 | 0.083 | 0.081 | 0.081 | 0.085 | 0.085 | 0.081 | 0.081 | 0.085 | 0.085 |
| **MTB_17** | 0.012 | 0.009 | 0.009 | 0.010 | 0.009 | 0.007 | 0.005 | 0.007 | 0.009 | 0.009 | 0.005 | 0.003 | 0.003 | 0.005 | 0.003 | 0.003 |  | 0.003 | 0.002 | 0.088 | 0.077 | 0.075 | 0.079 | 0.077 | 0.073 | 0.071 | 0.075 | 0.088 | 0.087 | 0.088 | 0.087 | 0.087 | 0.085 | 0.085 | 0.088 | 0.088 | 0.085 | 0.085 | 0.088 | 0.088 |
| **MTB_18** | 0.015 | 0.012 | 0.009 | 0.010 | 0.009 | 0.007 | 0.005 | 0.007 | 0.009 | 0.009 | 0.005 | 0.003 | 0.003 | 0.005 | 0.003 | 0.003 | 0.003 |  | 0.002 | 0.085 | 0.077 | 0.075 | 0.079 | 0.077 | 0.073 | 0.071 | 0.075 | 0.088 | 0.087 | 0.088 | 0.087 | 0.087 | 0.085 | 0.085 | 0.088 | 0.088 | 0.085 | 0.085 | 0.088 | 0.088 |
| **MTB_19** | 0.014 | 0.010 | 0.007 | 0.009 | 0.007 | 0.005 | 0.003 | 0.005 | 0.007 | 0.007 | 0.003 | 0.002 | 0.002 | 0.003 | 0.002 | 0.002 | 0.002 | 0.002 |  | 0.087 | 0.075 | 0.073 | 0.077 | 0.075 | 0.071 | 0.070 | 0.073 | 0.087 | 0.085 | 0.087 | 0.085 | 0.085 | 0.083 | 0.083 | 0.087 | 0.087 | 0.083 | 0.083 | 0.087 | 0.087 |
| **MTB_20** | 0.100 | 0.088 | 0.079 | 0.081 | 0.079 | 0.081 | 0.083 | 0.085 | 0.083 | 0.090 | 0.087 | 0.088 | 0.088 | 0.090 | 0.088 | 0.088 | 0.088 | 0.085 | 0.087 |  | 0.070 | 0.075 | 0.071 | 0.073 | 0.070 | 0.070 | 0.071 | 0.066 | 0.064 | 0.070 | 0.068 | 0.064 | 0.062 | 0.062 | 0.066 | 0.066 | 0.066 | 0.066 | 0.062 | 0.066 |
| **MTB_21** | 0.088 | 0.081 | 0.071 | 0.073 | 0.075 | 0.073 | 0.075 | 0.073 | 0.071 | 0.079 | 0.075 | 0.077 | 0.077 | 0.079 | 0.077 | 0.077 | 0.077 | 0.077 | 0.075 | 0.070 |  | 0.005 | 0.002 | 0.003 | 0.007 | 0.009 | 0.009 | 0.049 | 0.047 | 0.049 | 0.047 | 0.044 | 0.042 | 0.042 | 0.049 | 0.049 | 0.049 | 0.049 | 0.049 | 0.049 |
| **MTB_22** | 0.087 | 0.079 | 0.070 | 0.071 | 0.073 | 0.071 | 0.073 | 0.071 | 0.070 | 0.077 | 0.073 | 0.075 | 0.075 | 0.077 | 0.075 | 0.075 | 0.075 | 0.075 | 0.073 | 0.075 | 0.005 |  | 0.003 | 0.002 | 0.009 | 0.010 | 0.010 | 0.051 | 0.049 | 0.047 | 0.046 | 0.046 | 0.047 | 0.044 | 0.051 | 0.051 | 0.051 | 0.051 | 0.051 | 0.051 |
| **MTB_23** | 0.090 | 0.083 | 0.073 | 0.075 | 0.077 | 0.075 | 0.077 | 0.075 | 0.073 | 0.081 | 0.077 | 0.079 | 0.079 | 0.081 | 0.079 | 0.079 | 0.079 | 0.079 | 0.077 | 0.071 | 0.002 | 0.003 |  | 0.002 | 0.009 | 0.010 | 0.010 | 0.047 | 0.046 | 0.047 | 0.046 | 0.042 | 0.044 | 0.040 | 0.047 | 0.047 | 0.047 | 0.047 | 0.047 | 0.047 |
| **MTB_24** | 0.088 | 0.081 | 0.071 | 0.073 | 0.075 | 0.073 | 0.075 | 0.073 | 0.071 | 0.079 | 0.075 | 0.077 | 0.077 | 0.079 | 0.077 | 0.077 | 0.077 | 0.077 | 0.075 | 0.073 | 0.003 | 0.002 | 0.002 |  | 0.007 | 0.009 | 0.009 | 0.049 | 0.047 | 0.046 | 0.044 | 0.044 | 0.046 | 0.042 | 0.049 | 0.049 | 0.049 | 0.049 | 0.049 | 0.049 |
| **HAR_1** | 0.085 | 0.077 | 0.068 | 0.070 | 0.071 | 0.070 | 0.071 | 0.073 | 0.071 | 0.075 | 0.071 | 0.073 | 0.073 | 0.075 | 0.073 | 0.073 | 0.073 | 0.073 | 0.071 | 0.070 | 0.007 | 0.009 | 0.009 | 0.007 |  | 0.002 | 0.005 | 0.049 | 0.047 | 0.046 | 0.044 | 0.044 | 0.042 | 0.042 | 0.049 | 0.049 | 0.049 | 0.049 | 0.049 | 0.049 |
| **HAR_2** | 0.083 | 0.075 | 0.066 | 0.068 | 0.070 | 0.068 | 0.070 | 0.071 | 0.070 | 0.073 | 0.070 | 0.071 | 0.071 | 0.073 | 0.071 | 0.071 | 0.071 | 0.071 | 0.070 | 0.070 | 0.009 | 0.010 | 0.010 | 0.009 | 0.002 |  | 0.007 | 0.051 | 0.049 | 0.047 | 0.046 | 0.046 | 0.044 | 0.044 | 0.051 | 0.051 | 0.051 | 0.051 | 0.051 | 0.051 |
| **HAR_3** | 0.087 | 0.079 | 0.070 | 0.071 | 0.073 | 0.071 | 0.073 | 0.075 | 0.073 | 0.077 | 0.073 | 0.075 | 0.075 | 0.077 | 0.075 | 0.075 | 0.075 | 0.075 | 0.073 | 0.071 | 0.009 | 0.010 | 0.010 | 0.009 | 0.005 | 0.007 |  | 0.047 | 0.046 | 0.044 | 0.042 | 0.042 | 0.040 | 0.040 | 0.047 | 0.047 | 0.047 | 0.047 | 0.047 | 0.047 |
| **ANP_1** | 0.096 | 0.088 | 0.079 | 0.081 | 0.083 | 0.081 | 0.083 | 0.085 | 0.083 | 0.083 | 0.087 | 0.088 | 0.085 | 0.087 | 0.088 | 0.085 | 0.088 | 0.088 | 0.087 | 0.066 | 0.049 | 0.051 | 0.047 | 0.049 | 0.049 | 0.051 | 0.047 |  | 0.002 | 0.007 | 0.009 | 0.005 | 0.007 | 0.007 | 0.003 | 0.003 | 0.003 | 0.003 | 0.003 | 0.003 |
| **ANP_2** | 0.094 | 0.087 | 0.077 | 0.079 | 0.081 | 0.079 | 0.081 | 0.083 | 0.081 | 0.081 | 0.085 | 0.087 | 0.083 | 0.085 | 0.087 | 0.083 | 0.087 | 0.087 | 0.085 | 0.064 | 0.047 | 0.049 | 0.046 | 0.047 | 0.047 | 0.049 | 0.046 | 0.002 |  | 0.005 | 0.007 | 0.003 | 0.005 | 0.005 | 0.002 | 0.002 | 0.002 | 0.002 | 0.002 | 0.002 |
| **ANP_3** | 0.096 | 0.088 | 0.079 | 0.081 | 0.083 | 0.081 | 0.083 | 0.085 | 0.083 | 0.083 | 0.087 | 0.088 | 0.085 | 0.087 | 0.088 | 0.085 | 0.088 | 0.088 | 0.087 | 0.070 | 0.049 | 0.047 | 0.047 | 0.046 | 0.046 | 0.047 | 0.044 | 0.007 | 0.005 |  | 0.002 | 0.005 | 0.007 | 0.007 | 0.007 | 0.007 | 0.007 | 0.007 | 0.007 | 0.007 |
| **ANP_4** | 0.094 | 0.087 | 0.077 | 0.079 | 0.081 | 0.079 | 0.081 | 0.083 | 0.081 | 0.081 | 0.085 | 0.087 | 0.083 | 0.085 | 0.087 | 0.083 | 0.087 | 0.087 | 0.085 | 0.068 | 0.047 | 0.046 | 0.046 | 0.044 | 0.044 | 0.046 | 0.042 | 0.009 | 0.007 | 0.002 |  | 0.007 | 0.009 | 0.009 | 0.009 | 0.009 | 0.009 | 0.009 | 0.009 | 0.009 |
| **ANP_5** | 0.094 | 0.087 | 0.077 | 0.079 | 0.081 | 0.079 | 0.081 | 0.083 | 0.081 | 0.081 | 0.085 | 0.087 | 0.083 | 0.085 | 0.087 | 0.083 | 0.087 | 0.087 | 0.085 | 0.064 | 0.044 | 0.046 | 0.042 | 0.044 | 0.044 | 0.046 | 0.042 | 0.005 | 0.003 | 0.005 | 0.007 |  | 0.002 | 0.002 | 0.005 | 0.005 | 0.005 | 0.005 | 0.005 | 0.005 |
| **ANP_6** | 0.092 | 0.085 | 0.075 | 0.077 | 0.079 | 0.077 | 0.079 | 0.081 | 0.079 | 0.079 | 0.083 | 0.085 | 0.081 | 0.083 | 0.085 | 0.081 | 0.085 | 0.085 | 0.083 | 0.062 | 0.042 | 0.047 | 0.044 | 0.046 | 0.042 | 0.044 | 0.040 | 0.007 | 0.005 | 0.007 | 0.009 | 0.002 |  | 0.003 | 0.007 | 0.007 | 0.007 | 0.007 | 0.007 | 0.007 |
| **ANP_7** | 0.092 | 0.085 | 0.075 | 0.077 | 0.079 | 0.077 | 0.079 | 0.081 | 0.079 | 0.079 | 0.083 | 0.085 | 0.081 | 0.083 | 0.085 | 0.081 | 0.085 | 0.085 | 0.083 | 0.062 | 0.042 | 0.044 | 0.040 | 0.042 | 0.042 | 0.044 | 0.040 | 0.007 | 0.005 | 0.007 | 0.009 | 0.002 | 0.003 |  | 0.007 | 0.007 | 0.007 | 0.007 | 0.007 | 0.007 |
| **ANP_8** | 0.096 | 0.088 | 0.079 | 0.081 | 0.083 | 0.081 | 0.083 | 0.085 | 0.083 | 0.083 | 0.087 | 0.088 | 0.085 | 0.087 | 0.088 | 0.085 | 0.088 | 0.088 | 0.087 | 0.066 | 0.049 | 0.051 | 0.047 | 0.049 | 0.049 | 0.051 | 0.047 | 0.003 | 0.002 | 0.007 | 0.009 | 0.005 | 0.007 | 0.007 |  | 0.003 | 0.003 | 0.003 | 0.003 | 0.003 |
| **ANP_9** | 0.096 | 0.088 | 0.079 | 0.081 | 0.083 | 0.081 | 0.083 | 0.085 | 0.083 | 0.083 | 0.087 | 0.088 | 0.085 | 0.087 | 0.088 | 0.085 | 0.088 | 0.088 | 0.087 | 0.066 | 0.049 | 0.051 | 0.047 | 0.049 | 0.049 | 0.051 | 0.047 | 0.003 | 0.002 | 0.007 | 0.009 | 0.005 | 0.007 | 0.007 | 0.003 |  | 0.003 | 0.003 | 0.003 | 0.003 |
| **ANP_10** | 0.092 | 0.085 | 0.075 | 0.077 | 0.079 | 0.077 | 0.079 | 0.081 | 0.079 | 0.079 | 0.083 | 0.085 | 0.081 | 0.083 | 0.085 | 0.081 | 0.085 | 0.085 | 0.083 | 0.066 | 0.049 | 0.051 | 0.047 | 0.049 | 0.049 | 0.051 | 0.047 | 0.003 | 0.002 | 0.007 | 0.009 | 0.005 | 0.007 | 0.007 | 0.003 | 0.003 |  | 0.003 | 0.003 | 0.003 |
| **ANP_11** | 0.092 | 0.085 | 0.075 | 0.077 | 0.079 | 0.077 | 0.079 | 0.081 | 0.079 | 0.079 | 0.083 | 0.085 | 0.081 | 0.083 | 0.085 | 0.081 | 0.085 | 0.085 | 0.083 | 0.066 | 0.049 | 0.051 | 0.047 | 0.049 | 0.049 | 0.051 | 0.047 | 0.003 | 0.002 | 0.007 | 0.009 | 0.005 | 0.007 | 0.007 | 0.003 | 0.003 | 0.003 |  | 0.003 | 0.003 |
| **ANP_12** | 0.096 | 0.088 | 0.079 | 0.081 | 0.083 | 0.081 | 0.083 | 0.085 | 0.083 | 0.083 | 0.087 | 0.088 | 0.085 | 0.087 | 0.088 | 0.085 | 0.088 | 0.088 | 0.087 | 0.062 | 0.049 | 0.051 | 0.047 | 0.049 | 0.049 | 0.051 | 0.047 | 0.003 | 0.002 | 0.007 | 0.009 | 0.005 | 0.007 | 0.007 | 0.003 | 0.003 | 0.003 | 0.003 |  | 0.003 |
| **ANP_13** | 0.096 | 0.088 | 0.079 | 0.081 | 0.083 | 0.081 | 0.083 | 0.085 | 0.083 | 0.083 | 0.087 | 0.088 | 0.085 | 0.087 | 0.088 | 0.085 | 0.088 | 0.088 | 0.087 | 0.066 | 0.049 | 0.051 | 0.047 | 0.049 | 0.049 | 0.051 | 0.047 | 0.003 | 0.002 | 0.007 | 0.009 | 0.005 | 0.007 | 0.007 | 0.003 | 0.003 | 0.003 | 0.003 | 0.003 |  |

Table S3. MtDNA haplogroup population pairwise *F*_ST_ values. Produced with Arlequin ver 3.5.2.2.

|  | **MTB Haplogroup 1** | **Ovens Haplogroup** | **Kiewa Haplogroup** | **MTB Haplogroup 2** |
| --- | --- | --- | --- | --- |
| **MTB Haplogroup 1** | 0 | 0.92 | 0.94 | 0.94 |
| **Ovens Haplogroup** | 0.92 | 0 | 0.90 | 0.92 |
| **Kiewa Haplogroup** | 0.94 | 0.90 | 0 | 0.94 |
| **MTB Haplogroup 2** | 0.94 | 0.92 | 0.94 | 0 |

Figure S2. Maximum Likelihood Species Delineation Tree. Blue branches represent relationships between separate species, red branches represent relationships within the same species. Created in the Exelixis Lab bPTP web server (<https://species.h-its.org/ptp/>).

Figure S3. Full dataset PCA (Principal Component Analysis) plot. Individuals are plotted according to their relative genetic differences between one another, colours denote the site that the individual was collected from, n is the number of individuals collected from each site.


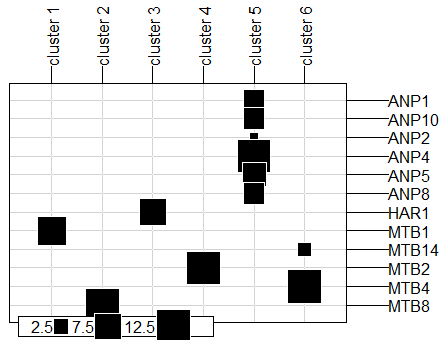


Figure S4. Full dataset DAPC Graph. Individual populations are grouped into clusters containing other closely related populations. Created with the adegenet package in R version 4.1.1.

Table S4. Fixed Difference Matrix representing the frequency of individual phylogenetic differences calculated between populations, with closely related populations sporting low values while greatly differentiated populations produced high values. Calculated with the dartR package in R version 4.1.1.

|  | **ANP1** | **ANP10** | **ANP2** | **ANP4** | **ANP5** | **ANP8** | **HAR1** | **MTB1** | **MTB14** | **MTB2** | **MTB4** | **MTB8** |
| --- | --- | --- | --- | --- | --- | --- | --- | --- | --- | --- | --- | --- |
| **ANP1** | 0 | 0 | 7 | 0 | 0 | 3 | 4668 | 2953 | 4642 | 2798 | 3214 | 3316 |
| **ANP10** | 0 | 0 | 19 | 1 | 0 | 1 | 3977 | 2412 | 3991 | 2266 | 2655 | 2764 |
| **ANP2** | 7 | 19 | 0 | 0 | 7 | 27 | 5161 | 3360 | 5104 | 3204 | 3624 | 3734 |
| **ANP4** | 0 | 1 | 0 | 0 | 0 | 4 | 4424 | 2783 | 4431 | 2621 | 3027 | 3128 |
| **ANP5** | 0 | 0 | 7 | 0 | 0 | 1 | 4757 | 3011 | 4705 | 2860 | 3278 | 3382 |
| **ANP8** | 3 | 1 | 27 | 4 | 1 | 0 | 4081 | 2503 | 4107 | 2369 | 2743 | 2865 |
| **HAR1** | 4668 | 3977 | 5161 | 4424 | 4757 | 4081 | 0 | 210 | 2213 | 329 | 949 | 1146 |
| **MTB1** | 2953 | 2412 | 3360 | 2783 | 3011 | 2503 | 210 | 0 | 278 | 2 | 26 | 79 |
| **MTB14** | 4642 | 3991 | 5104 | 4431 | 4705 | 4107 | 2213 | 278 | 0 | 226 | 25 | 83 |
| **MTB2** | 2798 | 2266 | 3204 | 2621 | 2860 | 2369 | 329 | 2 | 226 | 0 | 12 | 50 |
| **MTB4** | 3214 | 2655 | 3624 | 3027 | 3278 | 2743 | 949 | 26 | 25 | 12 | 0 | 5 |
| **MTB8** | 3316 | 2764 | 3734 | 3128 | 3382 | 2865 | 1146 | 79 | 83 | 50 | 5 | 0 |

Table S5. *F*_ST_ results produced between MTB and ANP SNP data with the hierfstat package in R version 4.1.1.

|  | **ANP1** | **ANP10** | **ANP4** | **ANP5** | **ANP8** | **HAR1** | **MTB1** | **MTB2** | **MTB4** | **MTB8** |
| --- | --- | --- | --- | --- | --- | --- | --- | --- | --- | --- |
| **ANP1** | NA | 0.1 | 0.036 | 0.03 | 0.16 | 0.88 | 0.7 | 0.7 | 0.71 | 0.74 |
| **ANP10** | 0.1 | NA | 0.15 | 0.13 | 0.085 | 0.85 | 0.67 | 0.68 | 0.69 | 0.71 |
| **ANP4** | 0.036 | 0.15 | NA | 0.051 | 0.23 | 0.9 | 0.77 | 0.76 | 0.76 | 0.79 |
| **ANP5** | 0.03 | 0.13 | 0.051 | NA | 0.19 | 0.89 | 0.72 | 0.71 | 0.72 | 0.75 |
| **ANP8** | 0.16 | 0.085 | 0.23 | 0.19 | NA | 0.85 | 0.67 | 0.68 | 0.69 | 0.72 |
| **HAR1** | 0.88 | 0.85 | 0.9 | 0.89 | 0.85 | NA | 0.39 | 0.49 | 0.58 | 0.63 |
| **MTB1** | 0.7 | 0.67 | 0.77 | 0.72 | 0.67 | 0.39 | NA | 0.13 | 0.28 | 0.37 |
| **MTB2** | 0.7 | 0.68 | 0.76 | 0.71 | 0.68 | 0.49 | 0.13 | NA | 0.23 | 0.33 |
| **MTB4** | 0.71 | 0.69 | 0.76 | 0.72 | 0.69 | 0.58 | 0.28 | 0.23 | NA | 0.2 |
| **MTB8** | 0.74 | 0.71 | 0.79 | 0.75 | 0.72 | 0.63 | 0.37 | 0.33 | 0.2 | NA |

Table S6. Bayes Factor Delimitation SNAPP model comparison table. Models are ranked based on Marginal Likelihood Estimate (MLE) and Bayes Factor (BF) (BF=2x(model1-model2)).

| **Model** | **No. of Species** | **MLE** | **BF** | **Rank** |
| --- | --- | --- | --- | --- |
| Harrietville, MTB, Kiewa_1, Kiewa_2, Kiewa_3 | 5 | -1471.2 | 67.2 | 3 |
| Kiewa: Harrietville: MTB_1: MTB_2 | 4 | -1461.0 | 46.8 | 2 |
| Kiewa: (Harrietville+MTB) | 2 | -1741.0 | 606.8 | 5 |
| Harrietville: Kiewa: MTB | 3 | -1494.7 | 114.2 | 4 |
| Kiewa_1: Kiewa_2: Kiewa_3: Harrietville: MTB_1: MTB_2 | 6 | -1437.6 | 0 | 1 |

Table S7. SNP-based Diversity tables. Ho = observed heterozygosity, He = expected heterozygosity, Hs = observed genetic diversity, Fis = inbreeding coefficient, and AR = Allelic richness. (a) All Sites, (b) Just MTB Sites, and (c) Just Kiewa Sites. Produced with the hierfstat and dartR packages in R version 4.1.1.

| **Site** | **Ho** | **He** | **Hs** | **Fis** | **AR** |
| --- | --- | --- | --- | --- | --- |
| ANP1 | 0.031 | 0.028 | 0.032 | 0.0034 | 1.03 |
| ANP10 | 0.051 | 0.050 | 0.057 | 0.058 | 1.06 |
| ANP4 | 0.029 | 0.029 | 0.030 | 0.042 | 1.03 |
| ANP5 | 0.029 | 0.026 | 0.029 | 0.0042 | 1.03 |
| ANP8 | 0.050 | 0.048 | 0.055 | 0.045 | 1.05 |
| HAR1 | 0.058 | 0.061 | 0.066 | 0.085 | 1.06 |
| MTB1 | 0.15 | 0.18 | 0.20 | 0.22 | 1.19 |
| MTB2 | 0.14 | 0.18 | 0.19 | 0.20 | 1.19 |
| MTB4 | 0.15 | 0.18 | 0.19 | 0.20 | 1.19 |
| MTB8 | 0.13 | 0.16 | 0.18 | 0.23 | 1.17 |

(a)

| **Site** | **Ho** | **He** | **Hs** | **Fis** | **AR** |
| --- | --- | --- | --- | --- | --- |
| MTB1 | 0.20 | 0.25 | 0.27 | 0.23 | 1.26 |
| MTB2 | 0.20 | 0.24 | 0.26 | 0.21 | 1.25 |
| MTB4 | 0.21 | 0.25 | 0.27 | 0.18 | 1.26 |
| MTB8 | 0.19 | 0.23 | 0.24 | 0.21 | 1.24 |

(b)

| **Site** | **Ho** | **He** | **Hs** | **Fis** | **AR** |
| --- | --- | --- | --- | --- | --- |
| ANP1 | 0.27 | 0.25 | 0.28 | 0.018 | 1.28 |
| ANP10 | 0.34 | 0.35 | 0.39 | 0.095 | 1.39 |
| ANP4 | 0.24 | 0.25 | 0.26 | 0.085 | 1.26 |
| ANP5 | 0.25 | 0.24 | 0.27 | 0.035 | 1.26 |
| ANP8 | 0.34 | 0.34 | 0.38 | 0.083 | 1.37 |

(c)


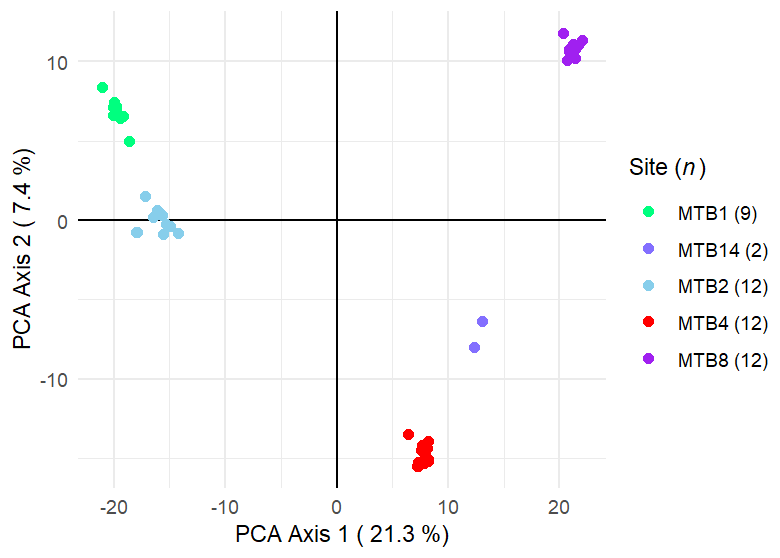


(a)


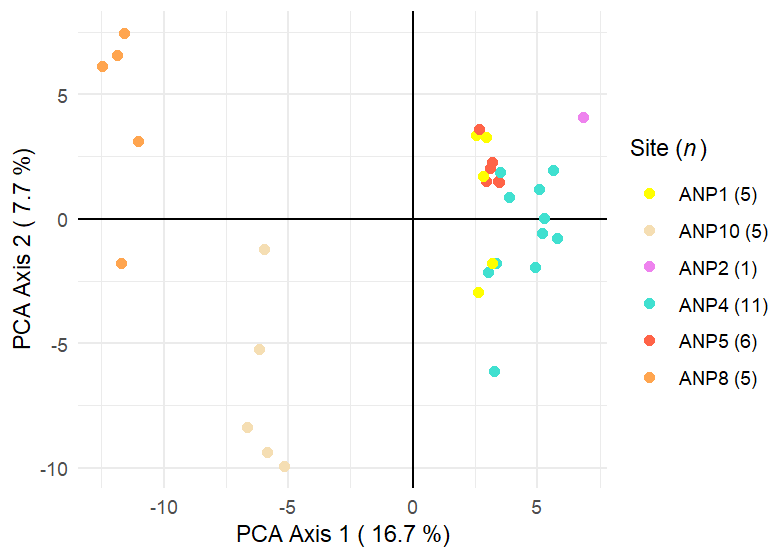


(b)

Figure S5. *Austrochloritis kosciuszkoensis* PCA (Principal Component Analysis) plots. Individuals are plotted according to their relative genetic differences between one another, colours denote the site that the individual was collected from, n is the number of individuals collected from each site. (a) just MTB samples, and (b) just Kiewa samples.


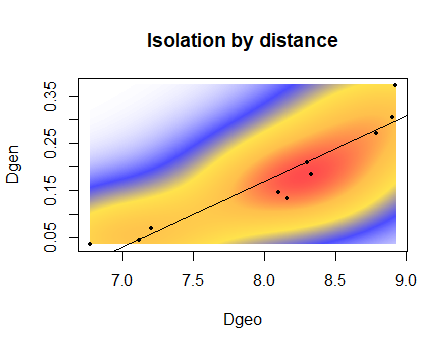


(a)


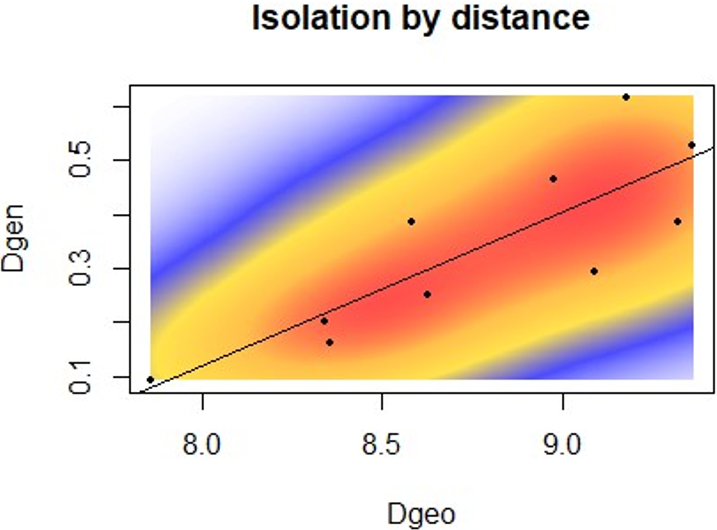


(b)

Figure S6. Isolation-by-distance graphs that visualise the relationship between the genetic distance (allele frequencies) and geographic distance (collection location in latitude and longitude) between individuals. Dgen represents genetic distance and Dgeo represents geographic distance. (a) Kiewa individuals (excluding ANP2), and (b) MTB individuals. Produced by the ade4 package in R version 4.1.1.


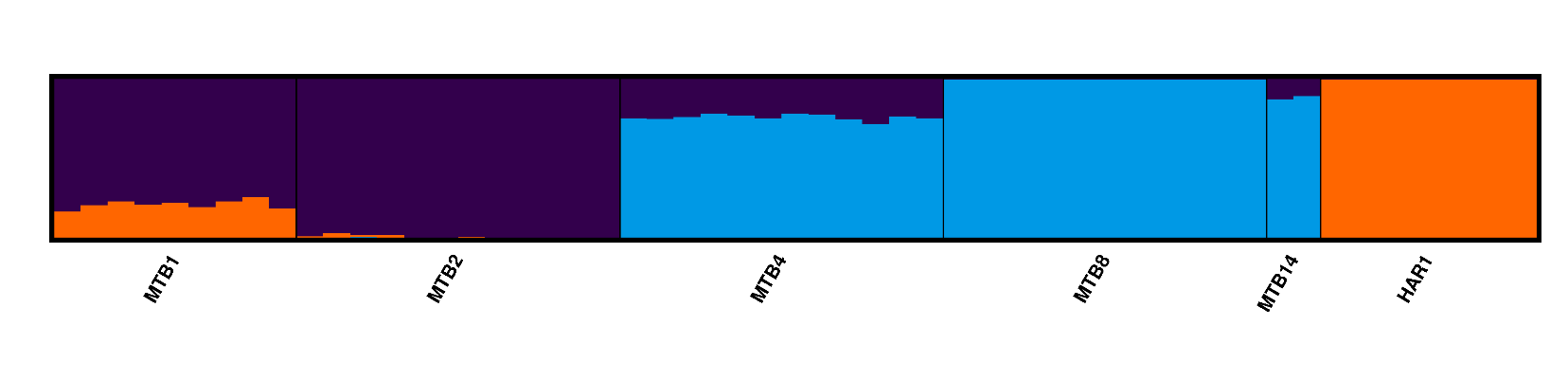


Figure S7. Individual proportions of Ancestry Coefficients of *Austrochloritis* sp. Mt Buffalo and Harrietville populations identified into clusters. Clusters are denoted by colour.
